# Formation of PLA Stereocomplex Crystals through Homorecrystallization and Mesophase Growth Mechanisms

**DOI:** 10.1021/acs.macromol.4c02836

**Published:** 2025-05-09

**Authors:** Hamid Ahmadi, Stan F.S.P. Looijmans, Marc P. F. H. L. van Maris, Pauline Schmit, Siavash Maraghechi, Patrick D. Anderson, Ruth Cardinaels

**Affiliations:** † Processing and Performance of Materials, Department of Mechanical Engineering, 418666 Eindhoven University of Technology, P.O. Box 513, MB Eindhoven 5600, The Netherlands; ‡ Multi-Scale Lab, Mechanics of Materials, Department of Mechanical Engineering, Eindhoven University of Technology, P.O. Box 513, MB Eindhoven 5600, The Netherlands; § Department of Chemical Engineering and Chemistry, 3169 Eindhoven University of Technology, P.O. Box 513, MB Eindhoven 5600, The Netherlands; ∥ Applied Mechanics, Department of Built Environment, 3169 Eindhoven University of Technology, P.O. Box 513, MB Eindhoven 5600, The Netherlands; ⊥ Soft Matter, Rheology and Technology, Department of Chemical Engineering, KU Leuven, Celestijnenlaan 200J, Leuven 3001, Belgium

## Abstract

The present study systematically investigates the crystallization kinetics and crystal morphology of polylactic acid (PLA) stereocomplex (SC) crystallization from the cold state in 1/1 poly l-lactic acid (PLLA) and poly d-lactic acid (PDLA) mixtures, i.e., via homorecrystallization and mesophase growth mechanisms at two different temperatures (190 and 215 °C). Thereto, homocrystals and mesophase precursors are generated at temperatures of 80, 100, and 120 °C. For the characterization of the crystalline structures, a combination of wide-angle X-ray diffraction (WAXD), small-angle X-ray scattering (SAXS), differential scanning calorimetry (DSC), and microscopy techniques is employed. The study reveals that homorecrystallization is much faster than crystallization via mesophase growth and can lead to the formation of either nodular-shaped stereocomplex crystals or coarser lamellar structures, depending on the crystallization temperature. The isothermal crystallization kinetics in a temperature jump stage is characterized via in situ WAXD and modeled with a modified Hillier-type model. The combination of thermal and structural characterization techniques allows to shed light on the factors that influence the crystalline structure of SC crystals, as well as the kinetics of their formation, which can be used for the development of high-performance PLA-based materials.

## Graphical abstract

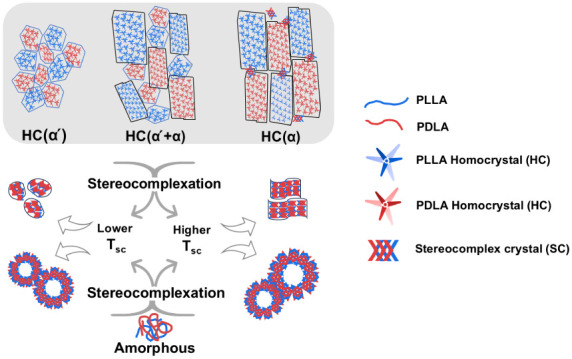

## Introduction

The development of biobased polymers has received increasing scientific and industrial interest as an effective solution to replace petroleum-based polymeric materials.^1^ Polymers from lactic acid are one of the main examples of these types of materials with the potential to be utilized in various applications such as commodity, pharmaceutical, and biomedical applications.^2^ However, owing to the low temperature resistance, toughness, and melt strength of PLA-based materials, their applications are severely limited. Many attempts have been made to improve the physical properties of PLA to meet manufacturing and end-use requirements.^3,4^ In particular, it has been proven that stereocomplex (SC) crystallization through blending of poly l-lactic acid (PLLA) and poly d-lactic acid (PDLA), i.e., through interchain interactions between two opposite enantiomeric l-lactide and d-lactide chains, provides an appropriate approach to improve the performance of PLA-based materials, including their mechanical properties,^5–9^ thermal stability,^10^ hydrolysis resistance^11–13^ and rheological properties.^14,15^

PLLA/PDLA racemic blends form diverse polymorphic crystalline structures, including stereocomplex crystals and different types of L and D homocrystals (HCs) (e.g., α′ and α types). SC crystals exhibit distinct structures and properties compared to α′- and α-type homocrystals. The chain packing in both the α′ and α types is similar, featuring an orthorhombic symmetry. In the ordered α-type structure, PLLA or PDLA chains adopt a 10_3_ conformation, tightly packed in an orthorhombic unit cell (dimensions: a = 1.08 nm, b = 0.620 nm, c = 2.88 nm, α = β = γ = 90°). The α′-type structure also displays orthorhombic symmetry, albeit with slightly larger lattice dimensions compared to the α-type.^16,17^ In contrast, SC crystals adopt a 3_1_ helical conformation with a triclinic unit cell structure (dimensions: a = 0.916 nm, b = 0.916 nm, c = 0.870 nm, α = 109.2°, β = 109.2°, and γ = 109.8°).^18^ Remarkably, the SC crystals possess a melting point of approximately 220 °C, which is approximately 50 °C higher than the melting point of the homocrystals.^19^

SC crystals can be generated upon cooling the PLLA/PDLA blend from the melt state, where the system is fully amorphous, or via cold crystallization, in which SC crystals are generated upon heating from a low temperature. Accordingly, two main mechanisms can lead to the formation of SC crystals. First, these crystals can originate from the amorphous region where PLLA/PDLA helix pairs convert to SC crystals via cluster formation, growth, and coalescence, i.e., the so-called mesophase growth (MG) mechanism.^20,21^ Second, in the context of cold crystallization, different types of homocrystals (α and α′) can also transform into SC crystals through melting and recrystallization at an elevated temperature below the melting point of the SC crystals, which is called homorecrystallization (HR).^22,23^

The ratio between PLLA and PDLA chains is one of the key factors determining the stereocomplexation of PLA-based materials. It has been shown that the maximum amount of SC crystals is formed in a 1:1 blend of PLLA and PDLA.^24^ To date, the SC crystallization of PLLA/PDLA 1:1 blends from the cold state where the L and D chains in the amorphous or homocrystalline phase of the blend are the SC crystal source, has been addressed extensively.^21,25–28^ Several studies have been executed to explain key parameters that affect the transition from homocrystals to SC crystals by using infrared spectroscopy and X-ray techniques.^22,29–33^ Fujita et al.^29^ studied the formation of SC crystals upon the reorganization of PLLA and PDLA single crystals. They demonstrated that in the course of this transition, chains or chain segments that detach from the homocrystal lattice remain aligned along the stacking direction, thereby facilitating the prompt growth of reorganized SC crystals that possess the same orientation. Additionally, they proposed that other chains or chain segments, which are not assimilated into the crystal lattice, may crystallize on the surface of surrounding crystals. Xiong et al.^32^ demonstrated that two populations of SC crystals form during the heating of PLLA/PDLA drawn film. They hypothesized that SC crystals which formed from initially oriented amorphous regions melt at a lower temperature, while the more stable SC crystals with higher melting temperatures might be those transformed from homocrystals. In a study, which set out to determine the effect of homocrystal type on SC crystallization, Na et al.^31^ clarified that the formation of SC crystals in 1:1 PLLA/PDLA blends is a complex process influenced by the nucleation density and segregation of PLLA and PDLA chains. The melting of small homocrystals from the α′-type leads to a high nucleation density, resulting in rapid SC formation and growth of rod-like crystals with low dimensions. In contrast, the melting of large homocrystals from the α-type leads to a low nucleation density and retarded SC formation, resulting in large plate-like and spherulitic aggregates.

The stereocomplex crystallization temperature (T_sc_) has been shown to alter the kinetics of stereocomplexation from the cold state, as well as the SC crystal structure, melting behavior, and crystalline size. Na et al.^31^ found that homorecrystallization at 200 °C exhibits a slower kinetics and results in larger SC crystals compared to that at 190 °C. Hu et al.^34^ demonstrated for solution-casted PLA that at stereocomplexation temperatures below 210 °C, the system exhibits three melting peaks, which they attributed to SC crystals originating from the amorphous, crystalline, and preordered states in the initial sample. However, for T_sc_ above 215 °C, the three melting peaks can no longer be observed. Yin et al.^33^ used SAXS and rheometry to investigate the SC crystallization behavior in racemic PLLA/PDLA during crystallization while heating and subsequent annealing at various temperatures. They observed a rather abrupt increase in the long period of SC crystals when the temperature exceeded 195 °C (from ∼ 18 and 19 nm at 185 and 195 °C to ∼ 40 nm at 215 °C), indicating the crucial role of chain diffusion between PLLA-rich and PDLA-rich domains. Their rheometry measurements revealed that at 185 or 195 °C, the system exhibited solid-like behavior, highlighting the role of SC crystals as physical cross-links in a PLA polymer melt. In contrast, at 225 °C, the measurements indicated liquid-like behavior. There is further evidence that the development of physical cross-links in the polymer melt originating from the initial SC crystallization between the L- and D-chains hinders chain diffusion and hence retards further SC crystallization.^35–37^

For homorecrystallization, it was suggested that SC crystals, formed at the interface between L- and D-type homocrystals, may form a barrier layer, thereby impeding chain diffusion and further SC crystallization.^29^ Previous studies have also suggested that SC crystals are formed due to the hydrogen-bonding interaction between enantiomeric polymers.^37^ Therefore, uniform mixing and good contact between PLLA and PDLA are required for the formation of SC crystals. However, the microscopic phase separation or demixing of PLLA/PDLA mixtures, especially with a high molecular weight (HMW), may lead to different SC crystallization behavior.^25,26^ Our hypothesis is that the mixing state of L and D chains can be affected by homocrystallization preceding SC formation. This occurs because homocrystallization induces chain ordering, which may create a favorable thermodynamic state for the subsequent formation of paired helices after melting the homocrystal. Overall, both the diffusion ability of the PLA polymer chains as well as their initial state of mixing play a crucial role in SC crystallization.

Thus, far, previous studies have shown that melting and recrystallization of homocrystals or mesophase growth are the main mechanisms of SC crystallization from the cold state. However, there has been little discussion about the effects of stereocomplex precursors and structure on the kinetics and microstructure of stereocomplexation in PLLA/PDLA 1:1 blends. Although Na et al.^31^ employed Polarized Optical Microscopy (POM) and Small-Angle Light Scattering (SALS) analyses for microscale characterization of the formed SC crystals through homorecrystallization, supplementing these methods with other techniques such as Wide-Angle X-ray Diffraction (WAXD), Small-Angle X-ray Scattering (SAXS), and Atomic Force Microscopy (AFM) would offer a more comprehensive understanding of the microstructural evolution during stereocomplexation from the cold state. In addition, due to potential conversions between different homocrystal types as well as SC mesophase or crystal formation, fast heating and cooling is required, which is challenging with traditional heating stages. The specific objective of the present research is to reveal the interplay between SC crystal sources, i.e., amorphous or homocrystalline phases, and their effects on the kinetics and microstructure of SC crystallization at different temperatures in 1:1 PLLA/PDLA blends by utilizing a jump-stage modified in-house, which enables the rapid transformation of SC sources to conditions conducive to stereocomplex formation. In particular, it is demonstrated that the different interfacial states between D- and L-homocrystal lamellae, which transform into SC crystals, especially in the early stages of stereocomplexation, control the kinetics and microstructure of the generated SC crystals. The microstructures of the generated SC crystals from various sources are characterized and compared by using a series of microstructure analysis techniques such as SAXS, WAXD, Differential Scanning Calorimetry (DSC), polarized microscopy, and AFM images to explain the relationship between the preconditioning process and the developed SC crystalline phase structure. Moreover, the primary and secondary crystallization kinetics of SC crystals, formed through melting and recrystallization of homocrystals or via mesophase growth, are analyzed using the in situ WAXD data and a modified Hillier type model, which is based on the Avrami model.

## Materials and Methods

### Materials and Sample Preparation

Poly L-lactic acid (Luminy-L175 with optical purity> 99%, *M*_w_ = 208 kg/mol, *M*_n_ = 87 kg/mol, polydispersity PDI = 2.41) and poly D-lactic acid (PDLA-D120 with optical purity >99%, *M*_w_ = 157 kg/mol, *M*_n_ = 79 kg/mol, polydispersity PDI = 1.99) were kindly provided by TotalEnergies Corbion (Gorinchem, The Netherlands).

PLLA and PDLA (weight ratio 1:1) were melt-blended under dry nitrogen flow at 240 °C with a rotation speed of 50 rpm for 10 min using a DSM Xplore micro 15 cc twin-screw compounder (DSM, Geleen, The Netherlands). A predetermined amount of dried PLLA/PDLA extruded blend was placed in a circular mold for compression molding to form disks with a diameter of 25 mm and thickness of 0.5 mm. All the materials and samples were dried in a vacuum oven at 40 °C for 24 h prior to processing and testing.

As listed by Tsuji, the melting points of SC crystals and homocrystals are reported to be in the ranges of 220–230 °C and 170–190 °C, respectively.^24^ Additionally, the equilibrium melting point of SC crystals is documented to fall between 250 and 279 °C, depending on the molecular weight of PLA.^38^ Conversely, the reported melting points for HCs are in the range of 205–215 °C. To generate various types of homocrystals and stereocomplex mesophases, the 1:1 blend of PLLA/PDLA was subjected to annealing at various temperatures (*T*_c_= 80 °C, 100 and 120 °C) and for different time intervals (1200 s to form α′-type HCs, 600 s to form α and α′+α type HCs, and 30 s to generate stereocomplex mesophase), as per the time–temperature protocol depicted in Figure 1a. As can be seen in Figure 1a, the different SC crystal sources made in step 1 are converted to SC crystals at two different temperatures (T_sc_= 190 and 215 °C) in step 2 and the SC crystals formed after a certain time are characterized using differential scanning calorimetry (DSC), polarized optical microscopy (POM) and atomic force microscopy (AFM) techniques. The precursor samples (obtained after step 1) are named as HC (α), HC­(α′+α), HC­(α′), and MP for respectively α, α′+α and α′ homocrystals or mesophase precursors whereas the samples containing SC crystals (obtained after step 2) are named as HR­(α), HR­(α′+α), HR­(α′) and MG. For the X-ray measurements, diverse SC crystal sources are generated at the cold stage of an in-house modified setup (Figure 1b, step 1 in Figure 1a) and subsequently transformed into SC crystals at the hot stage of the setup (step 2 in Figure 1b). A detailed description of this device is provided in the following section.

**Figure 1:**
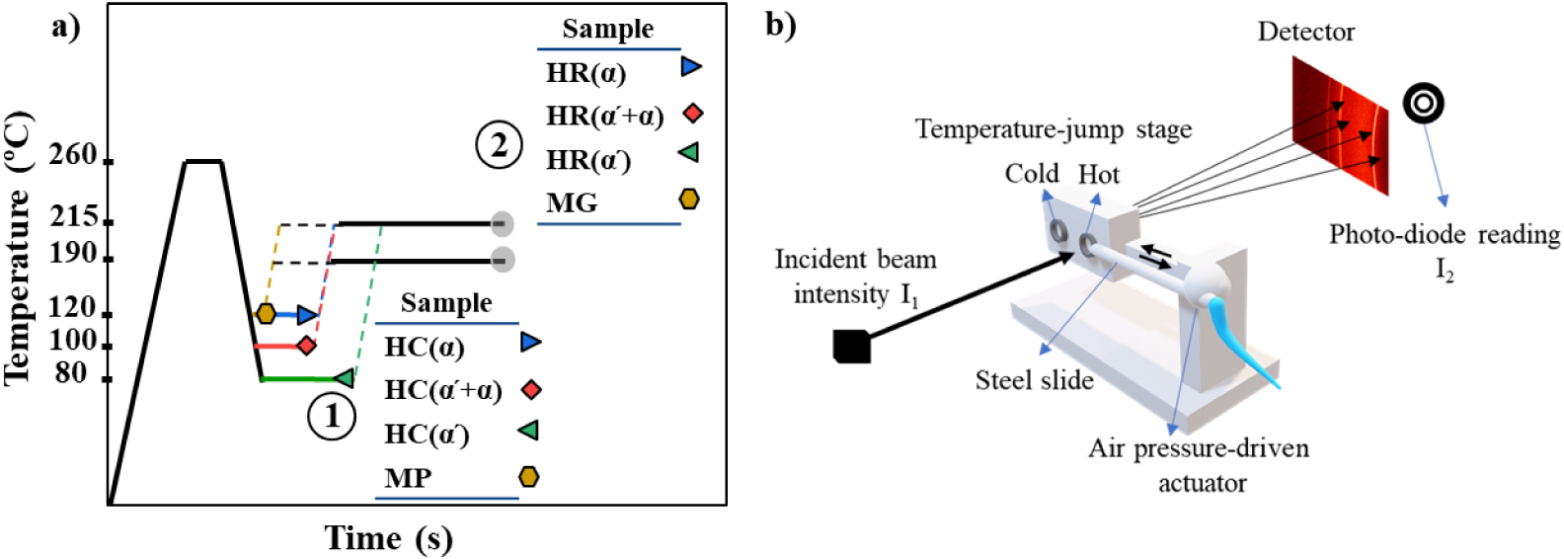
a) Thermal protocol employed to generate samples with various crystal types. b) Schematic of X-ray measurement setup.

### Characterization

In-situ X-ray measurements were carried out at the European Synchrotron Radiation Facility’s Dutch-Belgian beamline BM26 in Grenoble (France). Cut samples with a diameter of approximately 5 mm were sandwiched between two aluminum foils with a thickness of 20 μm. Samples were placed on a custom-modified JHT-350 temperature-jump stage (Linkam, Japan). The device consists of two heating stages with separate temperature control, allowing the sample to be quickly moved from one stage to the other, thereby experiencing a sudden temperature jump.^39^ A remotely controlled and air pressure-driven actuator, attached to the steel slide, added in-house to remotely move the samples back and forth between the stages at a repeatable speed, is used to apply the desired temperature jumps (Figure 1b). The cold stage is set at the desired temperature for making SC crystal sources (step 1: 80, 100, and 120 °C), and the hot stage is set at 190 or 215 °C (step 2). Initially, the sample is placed on the hot stage at 260 °C and kept at that temperature for 2 min to eliminate any thermal history. It is then transferred to the cold stage using a steel slide. Subsequently, the hot stage temperature is adjusted to either 190 or 215 °C, and the sample is moved from the cold stage to the hot stage after the preconditioning time has passed. A calibrated K-type microthermocouple coupled to a temperature readout device (VOLTCRAFT K202) was used to calibrate the temperature of both stages.

The experimental setup involved the use of X-ray radiation with a wavelength of 1.033 Å and a beam diameter of 300 μm × 400 μm, with scattering patterns being acquired over a 5 s exposure time. The samples were situated at distances of 28.5 cm and 3.44 m from the one-dimensional Wide Angle X-ray Diffraction (1D WAXD) Pilatus 300k detector with 172 μm × 172 μm pixel size (Dectris, Switzerland) and Small-Angle X-ray Scattering (SAXS) Pilatus 1 M detector with 172 μm × 172 μm pixel size (Dectris, Switzerland), respectively. The calibration of these detectors was conducted using Al_2_O_3_ (α-alumina) and AgBh (silver behenate) reference samples, respectively. The WAXD and SAXS intensities were subsequently determined through the use of azimuthal integration, after background subtraction, using the BUBBLE software,^40^ a fast azimuthal integrator based on the PyFAI python library. To enhance the precision of this process, a mask is applied to the data, thereby excluding pixel regions that correspond to the beam stop as well as any gaps inherent in the detector setup. The measured intensity (I_m_) and the air scattering background I_bkg_ are normalized by dividing them with a correction factor C, taking into account absorption and varying flux:

1 $$C=\frac{I_{1,\mathrm{bkg}}\cdot I_{2}}{I_{2,\mathrm{bkg}}\cdot I_{1}}$$

where *I*_1_ represents the incident flux, *I*_*1,bkg*_ the background incident flux, *I*_2_ the transmitted beam intensity, and *I*_*2,bkg*_ the transmitted beam intensity of the background. The transmitted intensity is measured by the photodiode placed in the beam stop of the SAXS detector. From the WAXD data, the crystallinity is determined by fitting the amorphous contribution with a Gauss-Lorentzian and the crystalline contributions with Lorentzian functions. The analysis of the SAXS data involved the calculation of a normalized one-dimensional correlation function defined as follows:^41^

$$\gamma_{1}\left(r\right)=\int_{0}^{\infty }I(q)q^{2}\cos\left(qr\right)dq/Q$$ 2

3 $$Q=\int_{0}^{\infty }I\left(q\right)q^{2}dq$$

where r represents the direction along which the electron density is measured and Q is the scattering invariant. Due to the finite range of experimentally accessible q values, the SAXS data needed to be extrapolated to both low and high q values. The Debye–Bueche model ( $I\left(q\to 0\right)=\frac{A}{{\left(1+q^{2}B^{2}\right)}^{2}}$ ) was used for extrapolation to zero q, where A and B are the constant and correlation length, respectively.^42^ The values of A and B were determined by curve-fitting the intensity data in the low q region (0.07–0.12 nm^–1^). The modified Porod model ( $I\left(q\to \infty \right)=\frac{K\exp\left(-\sigma^{2}q^{2}\right)}{q^{4}}+I_{B}$ ) was utilized for extending the SAXS data to high q values.^43^ In this model *K* is the Porod constant, σ is a parameter associated with the interface thickness between the crystal and amorphous phases, and *I*_*B*_ is the background scatter. The values of *K*, σ, and *I*_*B*_ were obtained by fitting the intensity profile at the high q region (0.2–1.6 nm^–1^) using a curve-fitting procedure.^44^

Differential scanning calorimetry (DSC) measurements were performed on a Mettler Toledo DSC823e, using sealed aluminum pans and specimens with a weight of 5–8 mg cut from hot-pressed samples. The DSC measurements were conducted following the time–temperature protocol illustrated in Figure 1a. First, the samples were molten at 260 °C for 2 min to erase the thermal history. Then, they were quickly cooled (50 °C min^–1^) to the homo crystallization temperatures (step 1: 80, 100, and 120 °C), and maintained at that temperature for the desired time to form homocrystals or mesophases, respectively. Afterward, the specimens were heated to the SC crystallization temperature (step 2: 190 or 215 °C) at 90 °C/min. Isothermal crystallization was allowed for various time durations whereby each isothermal cycle was then followed immediately by a heating scan at a rate of 10 °C/min.

The crystalline morphologies produced after the crystallization steps, as indicated in Figure 1a (depicting steps 1 and 2), were observed by polarized optical microscopy and atomic force microscopy. The polarized optical microscope utilized for observations was an Olympus BX51 model, which was equipped with a Charged-Coupled Device (CCD) digital camera and a custom-modified JHT-350 temperature-jump stage. The observations were conducted using a lens with a magnification of 20X on a sample that was placed between two thin cover glasses. Thin films with a thickness of approximately 30 μm were prepared by hot-pressing at 200 °C. Before imaging, the films were first subjected to annealing at 260 °C for 2 min on the hot stage to erase the thermal history, and subsequently transferred to the cold stage, which was aligned with the lens for imaging at the desired temperatures. Cryo-microtome slices of the quenched sample after crystallization (either only step 1 or steps 1 and 2 in Figure 1a) were examined using AFM (Digital Instrument Nanoscope IIIA Multimode) in tapping mode. Tapping silicon cantilevers (model ACTA-SS from APP NANO) with a nominal cantilever elastic constant of 25–75 N/m and a resonance frequency of 200–400 Hz and tip thickness of 1 nm (Figures 3 and 4) or 10 nm (Figures S4 and S7), were employed. Before the AFM measurements, the samples were sliced in a Leica RM2165 microtome equipped with a cryo-unit Leica LN21. The samples were cut using a diamond knife at – 20 °C and mounted in a special AFM holder.

## Results and Discussion

### Homocrystal and Mesophase Precursors

The formation of different homocrystal types is dependent on the crystallization temperature in step 1 (Figure 1a). The selected temperatures of 80, 100, and 120 °C are chosen based on the research of Zhang et al.^45^ and Wasanasuk et al.^46^ to generate α′, α′+ α and α types of HCs. Formation of the α′-type is favored by lowering the temperature.^47^ Previous studies have also suggested that due to the existence of various polymorphs in pure PLLA and racemic PLLA/PDLA blends, the crystallization kinetics exhibit a bimodal temperature dependence during melt crystallization, with the highest crystallization rates occurring at melt crystallization temperatures (*T*_c_) of 100 and 130 °C.^48,49^ Hence, to determine the optimal duration for the formation of distinct HC structures, the isothermal melt crystallization of this system is tested via calorimetry, and the results are presented in Figure S1. It is observed that the rate of crystallization is larger at a *T*_c_ of 100 and 120 °C, as compared to the *T*_c_ value of 80 °C. Therefore, the blend is annealed at 100 and 120 °C for 600 s to generate α′+α and α type of HCs, respectively. To produce a pure α′-type of homocrystals (HC) in the 1:1 PLLA/PDLA blends, the sample is annealed at 80 °C for a duration of 1200 s. During annealing, α′ homocrystals may transform to the α-type, even at low crystallization temperatures. The WAXD analysis of the sample crystallized at 80 °C for different times shows that an α′-to-α transformation occurs after t_c_ = 1200 s in this blend (Figure S2). Similar behavior has also been reported by Chen et al.^50^ They demonstrated that the α′-type is intrinsically metastable, and the α′-to-α transformation takes place in the later stages of crystallization at 80 °C. Therefore, annealing of the blend at 80 °C for 1200 s results in pure α′-type homocrystals.

It should be noted that at higher melt crystallization temperatures of the PLLA and PDLA blend, there is sufficient chain mobility for both stereocomplex and homocrystals to form concomitantly.^38^ Consequently, by subjecting the material to such a temperature for a shorter duration, it is possible to produce SC mesophases. Yang et al.^20^ demonstrated that the initial step in SC crystallization from the amorphous state involves the generation of paired 3_2_/3_1_ helices. They proposed that a subsequent intracluster ordering leads to the formation of clusters of helical pairs, referred to as the mesomorphic phase, which serve as nucleation sites. These nuclei continue to grow and eventually form SC crystals. Therefore, a sample was quenched from the melt and held at 120 °C for 30 s, resulting in the formation of mesophase, according to the above-mentioned mechanism.

The WAXD profiles generated concurrently with SAXS measurements of the homocrystals and mesophase precursors are presented in Figure 2. The WAXD results reveal the difference between the HC types generated at the different melt crystallization temperatures. The WAXD profiles of α′ and α crystals exhibit only minor differences, as depicted in Figure 2a. For the sample crystallized at 80 °C, the diffraction peaks located at q = 11.6, 13.2, and 17.3 nm^–1^ are attributed to the (110)/(200), (203), and (116) reflections of the α′-type HCs. However, the WAXD profile of the α-type HCs, obtained by crystallization at 120 °C, is characterized by a shift to higher q values of the (110)/(200) and (203) reflections, as well as an increase of the (010) peak intensity.^45,51^ Notably, the sample crystallized at 100 °C exhibits a combination of α′ and α types of HCs, as evident from the WAXD profile. Furthermore, compared to the WAXD profile of the samples annealed at 80 and 100 °C, the sample annealed at 120 °C displays additional reflections at q = 8.3, 14.6, and 16.9 nm^–1^, which are attributed to the reflections of the (110), (300/030), and (220) planes of SC crystals.^21^ Previous studies have demonstrated that SC crystals can form during homocrystallization at higher annealing temperatures, where sufficient molecular mobility is present.^38,52–55^ As shown in Figure 2a the mesophase formed at 120 °C does not show any obvious crystalline diffraction peak.

**Figure 2:**
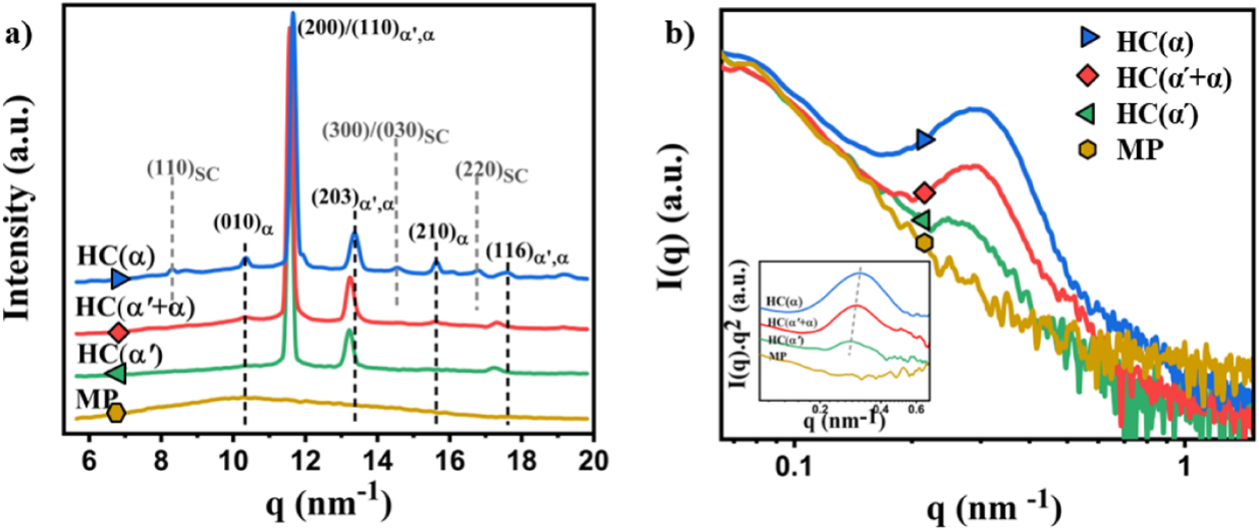
Representative a) 1D WAXD and b) SAXS curves of different types of homocrystals and mesophase precursors, prepared and measured at 80 °C, 100 °C, and 120 °C.

The crystallinity Χ_c_ is also calculated by deconvolution of the crystalline and amorphous peaks in the WAXD profile (Figure S3a-c). The obtained Χ_c_ values presented in Table S1 reveal a decrease in the amount of homocrystals with decreasing annealing temperature. For example, the mass fraction crystallinity of HC decreased from 38.4% for the α-type to 35.7% and 25.9% of homocrystals in the α′ + α and α′-type samples, respectively.

The SAXS patterns for the preformed samples are presented in Figure 2b, along with the corresponding Lorentz-corrected profiles (insert), the 1D correlation functions are shown in Figure S3d-f. It can be observed that the mesophase precursors do not display any noticeable scattering peak corresponding to a long period, due to the absence of a regular crystalline lamellar structure. However, the samples annealed at 80, 100, and 120 °C exhibit a single scattering peak in their respective SAXS profiles, corresponding to the scattering of the crystalline lamellae. The long period (L_p_), which is calculated as the first maximum in the 1D correlation function (eqs 2 and 3) from the SAXS profiles (Figure S3d-f), decreases from 20.8 to 18.9 nm as the melt crystallization temperature (*T*_c_) is increased from 80 to 120 °C (see Table S1). Additionally, α-type homocrystals formed at 120 °C possess the largest lamellar thickness, which was estimated from the 1D correlation function (Figure S3d-f) using the approach of Santa Cruz et al.^56^

The annealing temperature has also been found to affect the spherulite structure formed during the crystallization process. The effects of homocrystallization temperature on the spherulite morphology of PLLA/PDLA blends are studied using POM. The results, presented in Figure 3a-c, demonstrate that all samples exhibit spherulites with typical Maltese-cross patterns.^28^ These images show that decreasing the *T*_c_ leads to a decrease in spherulitic size in the PLLA/PDLA blend. The α′-type homocrystals, generated at 80 °C, exhibit the lowest spherulite size in comparison to the other samples. This phenomenon can be attributed to the higher supercooling and corresponding nucleation density at this temperature.^57^ Representative AFM phase images of PLLA/PDLA crystallized isothermally at 120 °C are also shown in Figure 3d,e (the AFM images for HC­(α′) and HC­(α′+α) are presented in Figure S4). As can be seen in Figure 3d, compact spherulitic structures are observed on the sample surface of HC­(α). In this sample, the HC lamellae are planar, while a grain-shaped structure is observed for HC­(α′) and HC­(α′+α) (Figure S4), which is consistent with the literature.^58–61^

**Figure 3:**
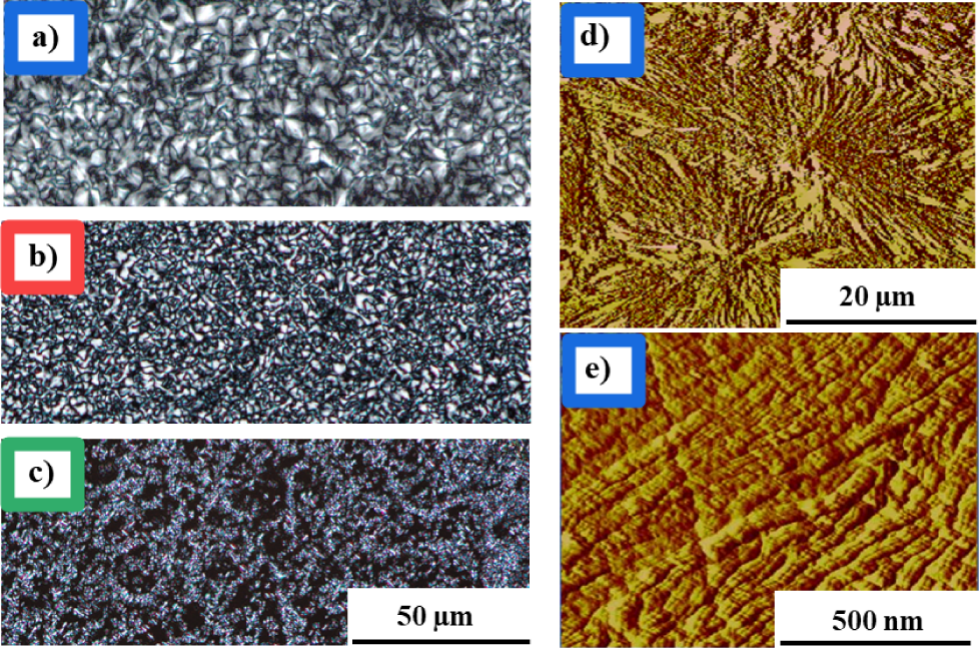
POM micrographs of different types of homocrystals, prepared at a) 120 °C and 600 s for HC­(α), b) 100 °C and 600 s for HC­(α′+α), c) 80 °C and 1200 s for HC­(α′) and AFM phase images of HC­(α) prepared at 120 °C and 600 s at different magnifications (d,e). The scales of all POM images are the same.

### Choice of the SC Crystallization Temperature (*T*_sc_)

The kinetics of formation, total amount and structure of SC crystals developed from the cold state depend on the SC crystallization temperature. Previous studies already noticed significant differences in the crystallization kinetics,^31^ melting behavior,^34^ long period of the SC crystals and rheology^33^ above and below a certain transition temperature, situated in the range of 200 °C - 215 °C. We assess differences in the formed SC crystals depending on the SC crystallization temperature (T_sc_, step 2) by evaluating the nonisothermal crystallization of the blend after cooling from this SC crystallization temperature. The peak crystallization temperature (*T*_cPeak_) and the enthalpy of crystallization, representing the total amount of crystals obtained during the cooling cycle are plotted against T_sc_ (Figure S5b-d). Both quantities show a clear two-step decrease with increasing T_sc_. These findings suggest that stereocomplexation at elevated temperatures, where only SC crystals can form, coming from the cold state, results in distinct SC crystallization behavior, particularly below 200 °C and above 210 °C. Hence, temperatures of 190 and 215 °C are chosen in the remainder of this work as the cold crystallization temperatures for SC crystallization (T_sc_ in step 2 in Figure 1a). The stereocomplexation behavior at these temperatures will be explored, encompassing the analysis of crystal structure and kinetics, starting from different precursor types.

### Effect of Preconditioning on Stereocomplexation at Different Temperatures

Figure 4a-c show POM images acquired after 30 min stereocomplexation from mesophases and 120 min SC crystallization starting from α-type homocrystals. It can be seen that as a result of the mesophase growth mechanism, a mesophase nucleus triggers further crystallization of the amorphous portion, giving rise to well-defined spherulitic structures (Figure 4a). This phenomenon bears resemblance to the homo crystallization process from the melt state (Figure 3a). The 30 min stereocomplexation time is selected for the mesophase growth sample since extensive crystalline growth at long times such as 120 min poses challenges to distinguish the spherulite shape from POM images due to the space-filling characteristics, as shown in Figure S6 and Movie S1. In Figure 4b,c representative images of HR­(α) at 190 and 215 °C are presented. The images depict the disappearance of the spherulitic structure of the HCs and the emergence of small aggregated structures of SC crystals. The transition from HC­(α′) and HC­(α′+α) samples to SC crystals, i.e., HR­(α′) and HR­(α′+α), shows a similar behavior (Figure S6 and Movies S2, S3, and S4).

**Figure 4:**
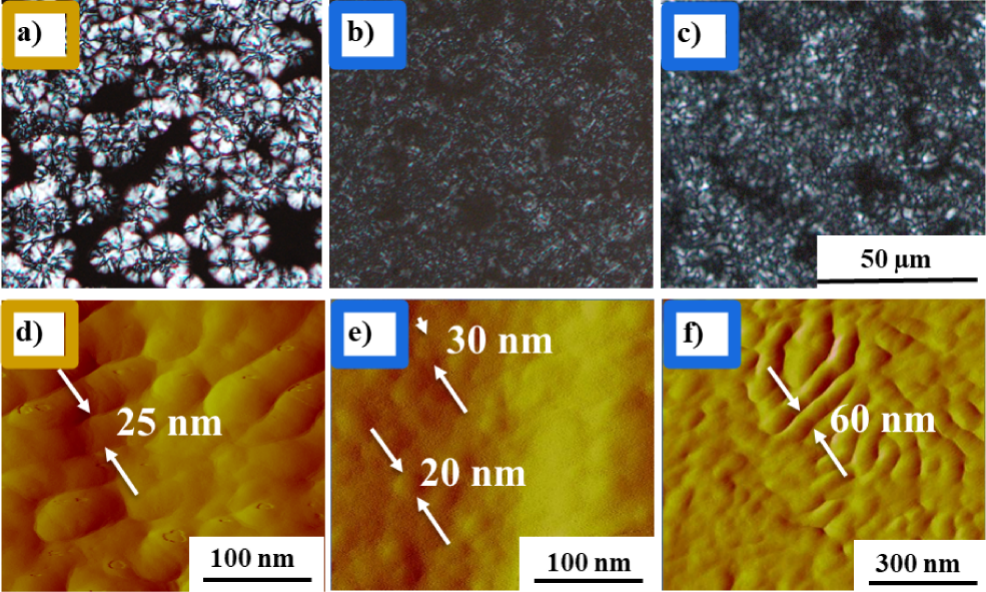
POM micrographs and AFM phase images of SC crystals formed from different sources and at different isothermal crystallization temperatures, a) Mesophase growth (MG) at 190 °C after t_c_ = 30 min (POM), b) HR­(α) at 190 °C after t_c_ = 120 min (POM), c) HR­(α) at 215 °C after t_c_ = 120 min (POM). d) Mesophase growth (MG) at 190 °C after t_c_ = 120 min (AFM), e) HR­(α) at 190 °C after t_c_ = 120 min (AFM), f) HR­(α) at 215 °C after t_c_ = 120 min (AFM).The scales of all POM images are the same.

The utilization of AFM offers the ability to visualize the surface morphology of SC crystals generated from different sources. As demonstrated in Figure 4d-f, the AFM phase images give a magnified view of the structure of the MG sample at 190 °C and the HR­(α) sample at 190 and 215 °C (the AFM images of HR­(α′) and HR­(α′+α) at different T_sc_ are presented in Figure S7). As shown in Figure 4a, stereocomplexation through mesophase growth results in a spherulitic shape. Figure 4d displays the AFM image of the ordered SC lamellae formed under this condition. However, homorecrystallization at 190 °C leads to SC crystals with a nodular shape (see Figures 4e and S7a,b). As previously noted, the polarized optical microscopy representation of the SC crystals generated through homorecrystallization at 190 °C evinces a conspicuous absence of spherulitic birefringent entities, as illustrated in Figure 4b. The AFM depiction of the identical specimen (Figure 4e) reveals a nodular shape morphology with an average dimension of 20–30 nm for this sample. This discernible behavior has been reported for various polymeric systems that crystallize at low temperatures or under high supercooling conditions, leading to the production of nonlamellar domains resembling discrete particles, devoid of higher-order superstructures such as spherulites.^62–64^ It is believed that the impediment of large scale lamellae with ordered structure stems from the low chain mobility caused by high supercooling or a low crystallization temperature, i.e., high nucleation rates, leading to rapid space filling.^57^ In comparison to the HR­(α) sample at 190 °C, SC crystals formed by melting of HCs at 215 °C have a more regular and coarser structure with larger domains (see Figures 4f and S7c,d). In this case, the formation of longer and more ordered lamellae may be explained by the higher chain mobility, which is related to the lower viscosity of the surrounding amorphous structure, and the higher mobility of the paired helices created following melting of D and L types of HCs at this temperature.

In order to provide quantitative information regarding the crystal structures and amount of SC crystals generated after 120 min at 190 and 215 °C from different sources, a combination of WAXD and SAXS data are analyzed, as presented in Figure 5. A comparison of the crystallization temperature effect as well as the effects of preconditioning on the amount of SC crystals after 120 min crystallization is presented in Figure 5a. The one-dimensional WAXD intensity profiles shown in Figure 5a reveal the presence of three distinct peaks located at q = 8.22, 14.4, and 16.7 nm^–1^, corresponding to the (110), (300/030) and (220) planes of the SC crystals.^20^ It should be noted that the content of SC crystals (Χ_c_) was determined by deconvoluting the WAXD pattern into two broad amorphous peaks and the crystalline contributions. A fit of the amorphous and crystalline peaks for a representative HR­(α) sample is summarized in Figure S8. It can be seen that homorecrystallization leads to an increased formation of SC crystals as compared to mesophase growth at the various crystallization temperatures. Furthermore, the findings indicate that homorecrystallization at 215 °C results in a higher amount of SC crystallinity which is attributed to the enhanced chain mobility that exists at this temperature. For the mesophase growth sample at 215 °C, the amount of SC crystals formed after 120 min is extremely low. Interestingly, homorecrystallization of HC­(α′+α) for 120 min results in a slightly higher percentage of SC crystals than for the other HC types at the same crystallization temperature, mainly at 190 °C.

**Figure 5:**
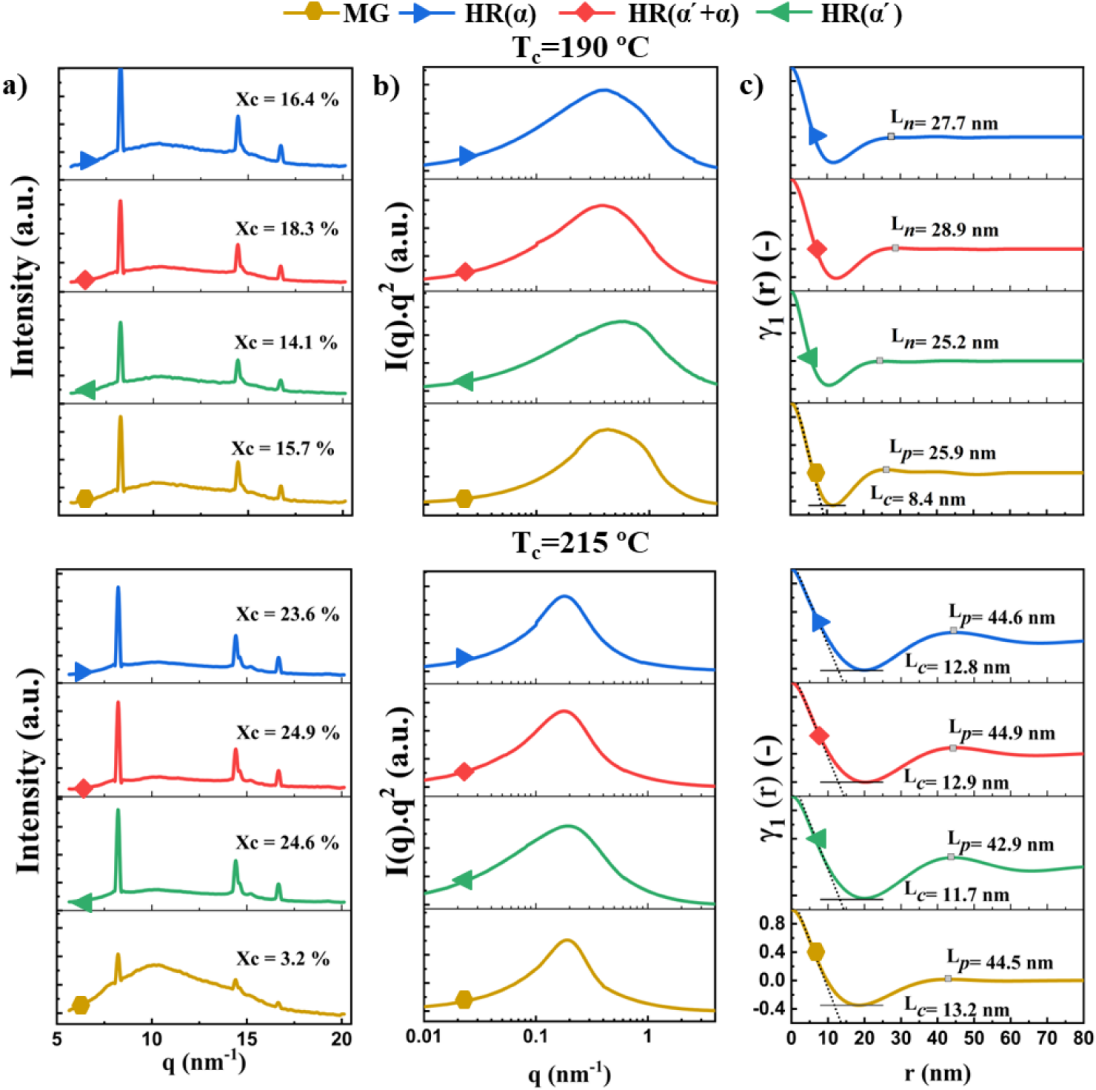
a) 1D WAXD patterns, b) Lorentz corrected SAXS profiles and c) Normalized correlation functions of SC crystallized from different sources for 120 min at 190 and 215 °C. The amount of crystallinity (Χ_c_), internodular distance (L_n_), long period (L_p_) and lamellar thickness (L_c_) are indicated on the plots.

The SC crystalline lamellar structure crystallized at different temperatures is also investigated via SAXS. The Lorentz-corrected SAXS profiles of samples crystallized at 190 and 215 °C for 120 min are presented in Figure 5b. At the lower crystallization temperature, a broader SAXS profile is observed, indicating the presence of SC crystals with various sizes and shapes. As the stereocomplexation temperature rises, the SAXS peak narrows and becomes more symmetrical. These observations suggest that the SC crystallization at the higher temperature of 215 °C yields a more structurally uniform SC crystalline lamellar phase. Furthermore, the Lorentz-corrected SAXS data reveal that the SC crystals derived from the α′-type HCs exhibit a broader peak at both 190 and 215 °C when compared to the other samples (Figure 5b). This can be attributed to the presence of crystalline domains with a larger size distribution in these samples. To ascertain the morphological parameters of the formed SC crystals, the obtained Lorentz-corrected SAXS profiles are subjected to an inverse-cosine Fourier transformation (eqs 2 and 3), resulting in a one-dimensional correlation function. It should be noted that the first maximum of the correlation function is designated as the internodular distance (L_n_) for SC crystals generated through homorecrystallization at 190 °C. Conversely, for the other samples, given the presence of an ordered structure resembling crystal lamellae, the long period (L_p_) and lamellar thickness (L_c_) are extracted. The structural parameters obtained from the one-dimensional correlation function are depicted in Figure 5c. The results show that the L_n_ values calculated for the SC crystals generated from different preformed HCs and the L_p_ value of MG at 190 °C range between 25 and 30 nm, which coincides with the internodular distances and domain width measured from AFM data (Figure 4d,e). However, as the crystallization temperature is increased, a more coarse and ordered lamellar structure is generated, resulting in a more clear maximum in the 1D correlation function corresponding to L_p_ and L_c_ values of 40–45 nm and 10–15 nm, respectively. Similarly, Yin et al.^33^ have reported nearly identical L_p_ values when SC crystals were produced after heating at 5 °C/min from the glassy state to 185 and 225 °C followed by 30 min annealing. Moreover, the results indicate that smaller SC crystals are formed when homorecrystallization of the α′-type is performed, as compared to the other samples. The formation of tiny SC crystals formed upon melting and recrystallization of HCs formed at 80 °C, i.e., α′-type, has also been reported before.^31^

The analysis of the melting behavior of the crystallized samples after homorecrystallization or mesophase growth can provide further information about the crystalline structure. Figure 6 illustrates the melting behavior of SC crystals obtained from diverse sources, each subjected to crystallization for a duration of 120 min at both low and high stereocomplexation temperatures. The presence of one single melting peak for the mesophase-grown SC sample at both crystallization temperatures corroborates the conclusion that this particular mechanism leads to the formation of uniform SC lamellae possessing identical thickness, which agrees with the SAXS results. Additionally, it is evident that the melting peak of the MG sample is situated at a higher temperature compared to that of SC crystals formed through homorecrystallization at the same temperature, indicative of the formation of SC lamellae with a larger lateral dimension and higher thickness. A different melting behavior is observed for the SC crystals formed via homorecrystallization, which can be seen in Figure 6a,b. These crystals display multiple melting peaks, irrespective of the HC source and T_sc_.

**Figure 6:**
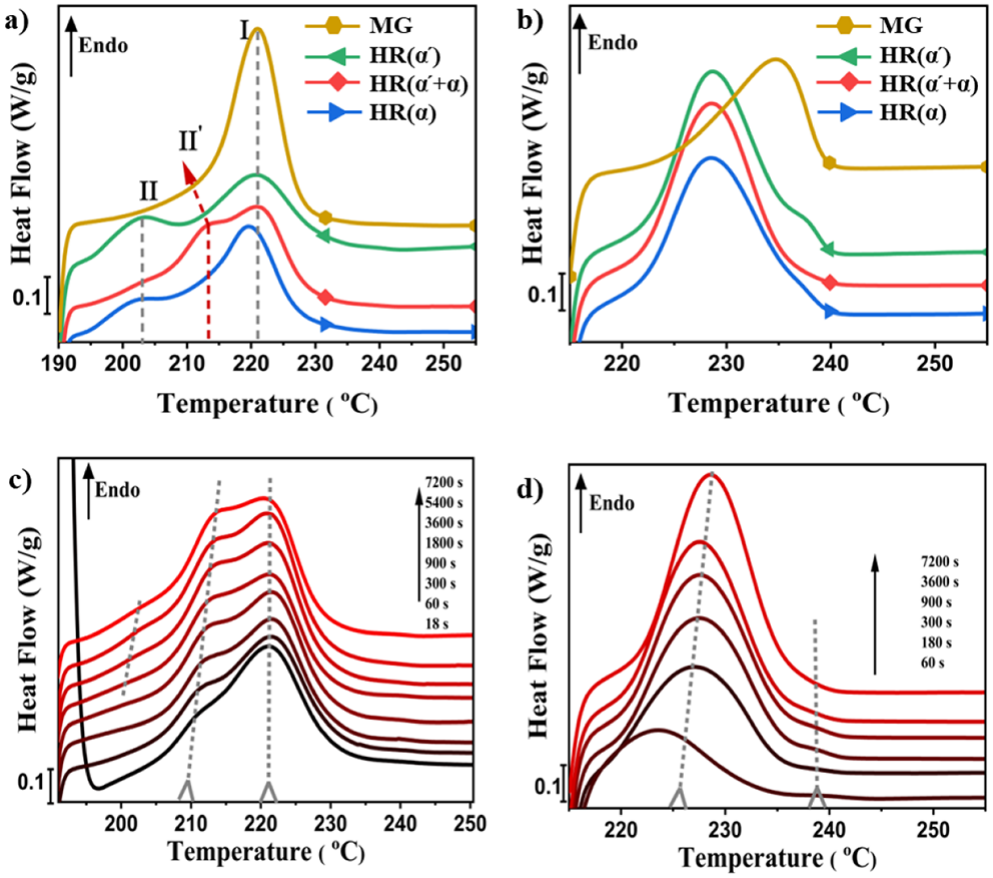
Melting behavior of SC recrystallized from different sources for 120 min at a) 190 °C, b) 215 °C. Evolution of the melting behavior after recrystallization of HC­(α'+α) for various times at c) 190 °C, d) 215 °C, all measured during heating at 10 °C/min.

The melting curves of HR­(α) and HR­(α′) samples at 190 °C exhibit two distinct endothermic peaks labeled as I and II (Figure 6a). In addition, it can be seen that upon scanning the melting of SC crystals formed by homorecrystallization of a mixture of α′ and α HCs at 190 °C, a new endotherm appears between the endothermic peaks I and II, which is denoted by II’. It can be argued that the presence of multiple melting peaks can be explained by a mechanism of melting-reorganization during heating.^65,66^ To provide further clarification, heating traces of the given samples are recorded at varying heating rates. Figure S9 illustrates the melting behavior of HR­(α) crystallites and mesophase grown SC crystals at heating rates of 5, 10, 20, and 30 °C/min. The occurrence of multiple melting peaks appears to be relatively unaffected by the heating rate. This indicates that there is no significant melting-reorganization during the melting process for the homorecrystallized SC crystals. Hence, the peaks II and II’ correspond to SC crystals formed under more restricted mobility, resulting in more imperfect lamellae.

To provide further information about the SC structures, we also analyzed the melting behavior after SC crystallization at 190 and 215 °C for various times. To do so, the samples are heated to 190 and 215 °C and crystallized at these temperatures for different periods of time following the formation of various types of HCs and mesophases. Subsequently, the samples are reheated at a rate of 10 °C/min to 260 °C to study the melting behavior of the generated SC crystals. Figure 6c,d depict the melting behavior corresponding to the HR­(α′+α) at 190 and 215 °C for varying time periods, respectively. The melting curves of SC crystals formed at 190 °C reveal that the location of the higher melting endotherm (I) remains almost unaltered regardless of the duration of stereocomplexation. This behavior can also be seen in the other samples as illustrated in Figure S10. However, it is observed that endothermic peak II’ occurs prior to endothermic peak II for the HR­(α′+α), and both peaks exhibit a shift toward higher temperatures with increasing crystallization time. The shifting of melting peak II to higher temperatures can also be seen for homorecrystallization of pure α′and α type HCs (Figure S10). The results of melting the SC crystals from HR­(α′+α) at 215 °C, where higher chain mobility is available, reveal the presence of two melting peaks. Similar to what was observed at 190 °C, the melting peak at the higher temperature (I) saturates and remains consistent after an initial period of crystallization. Meanwhile, the lower melting peak (II) grows and shifts to higher temperatures over time. This observed behavior is consistent across the HR­(α′) and HR­(α) at 215 °C (Figure S10).

Considering the different time point of occurrence of the melting peaks, it can be concluded that the high temperature endothermic peak can be attributed to the melting of thick lamellae or nodules that originated during the initial stages of SC crystallization, whereas the low temperature endothermic peaks can be associated with the proliferation of SC crystals formed during the later stages of crystallization. The appearance of peak II’ at a higher temperature than peak II suggests that the combination of two HC crystal types, each with their own size and melting dynamics, provides a favorable environment for the formation of more organized SC lamellae, leading to the observed shift in the melting temperature. The shifting of the lower melting peaks toward higher temperatures with increasing crystallization time suggests the occurrence of certain perfectioning mechanisms for SC crystals during the late stages of homorecrystallization.

### In-Situ 2D-WAXD Analysis during SC Formation

As explained in the materials and methods section, SC crystallization is investigated while applying a temperature profile consisting of two distinct steps (Figure 1a). First, the samples undergo annealing on the cold stage of the jumpstage where mesophase and various HC crystal types are generated, and are then instantly moved to the hot stage to generate SC crystals. After the annealing process on the cold stage and transfer of the samples to the hot stage, the WAXD patterns of the samples are recorded to evaluate the changes in crystal structure and crystallinity. Figure 7 presents the 2D WAXD patterns of the PLLA/PDLA blends after being transferred to the hot stage at 190 °C. Figure 7a-c display distinct reflections in the first WAXD pattern at *t* = 0, indicating that different crystalline structures developed during annealing at the various preconditioning temperatures. As time progresses, these reflections diminish rapidly, and the characteristic patterns of homocrystals disappear. Notably, after 11 s, only SC crystals are evident in the samples. Moreover, an interesting observation is made in the reflection at t_c_ = 6s for the homorecrystallization processes. The α-type HCs reflection starts to disappear at t_c_ = 6s (Figure 7a), while for the sample preformed at 80 °C (i.e., α′-type), the reflections of the α′-type are changed into those of the α-type before SC crystal formation (Figure 7c). This behavior is less prominent in the homorecrystallization of HC­(α′+α). The transition from the α′-type to the α-type is known to occur in the temperature range of 150–160 °C, which is close to the melting point of the HCs. This transition can be driven by several mechanisms, including solid–solid transformation and melting–recrystallization.^67–69^ The evolution of mesophases during SC crystallization at 190 °C can also be seen in Figure 7d, the lack of reflections in the first recorded 2D-WAXD image implies the absence of a crystalline structure in this sample and suggests direct formation of SC crystals from the amorphous state.

**Figure 7:**
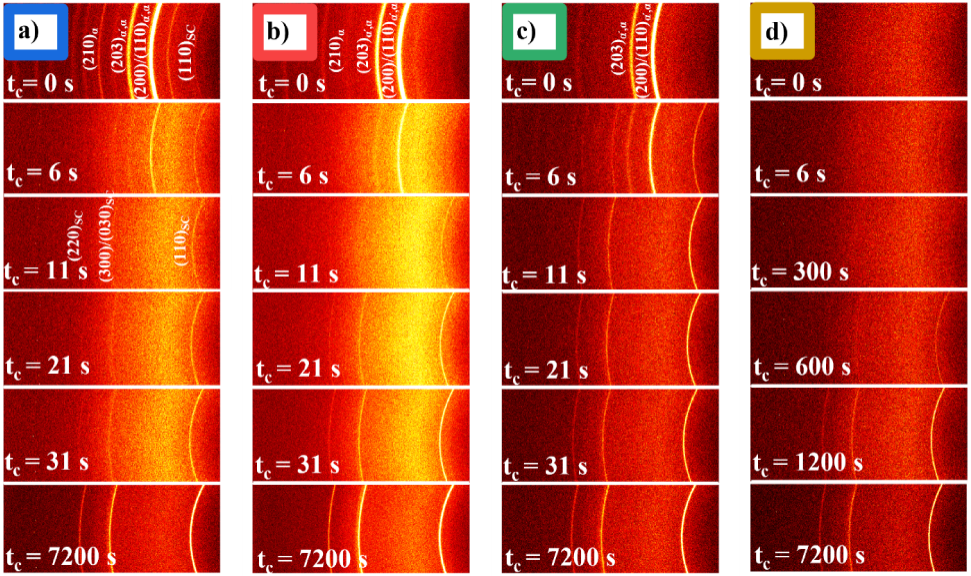
2D WAXD patterns of the SC crystals recrystallized at 190 °C from different sources, indicated time corresponds to time at 190 °C. a) Homorecrystallization from HC­(α), b) Homorecrystallization from HC­(α′+α), c) Homorecrystallization from HC­(α′), d) Mesophase growth.

In addition to the structural evolution during stereocomplex crystallization, distinct crystallization kinetics can be observed when starting from different HC sources. Figure S11 illustrates the corresponding 1D WAXD patterns of each sample at the specified times at 190 °C. Differences in the SC crystallinity generated at the same time can be seen across the different samples, indicating diverse crystallization kinetics. In the following section, the kinetics of SC crystallization by homorecrystallization from various HCs and through mesophase growth at 190 and 215 °C are discussed in detail.

### Time Dependence of Isothermal Crystallization via WAXD Analysis

The time-dependent growth of SC crystallinity during isothermal crystallization, initiated from different sources, at 190 and 215 °C, is illustrated in Figure 8. As pointed out before, the crystallinity is determined by deconvolution of the 1D WAXD profiles into amorphous and crystalline contributions (Figure S8). Figure 8a shows the kinetics of stereocomplexation at 190 °C. The graph displays a two-stage crystallization pattern on a logarithmic time axis, comprising of a sigmoid followed by a curve with a constant slope. For the stereocomplexation at 215 °C a very long induction time can be seen in Figure 8b. At T_sc_ = 190 °C, SC crystals from homorecrystallization begin to appear, and their quantity increases, from approximately 10 s after the temperature jump. In contrast, SC crystals from the mesophases only start to grow approximately 5 min after the temperature jump. A similar trend is observed for stereocomplexation at 215 °C, albeit with a longer crystallization time compared to that at 190 °C. A comparable trend is also evident from the DSC data obtained by melting the sample after different crystallization times at 190 and 215 °C (Figure S12). Although the final crystallinities obtained by both methods are similar, it should be noted that a relatively high SC crystallinity is already obtained after a short crystallization time at 190 and 215 °C compared to the WAXD measurements. This can be attributed to the low cooling and heating rates applied by the DSC. Consequently, SC crystals already form during heating, thereby making the initial stages of SC crystallization inaccessible in DSC studies. This is clearly demonstrated in Movie S1, which shows SC crystallization by mesophase growth, following either the thermal protocol of the DSC or that of the WAXD study. When using the DSC thermal protocol, crystallization is initiated earlier during heating.

**Figure 8:**
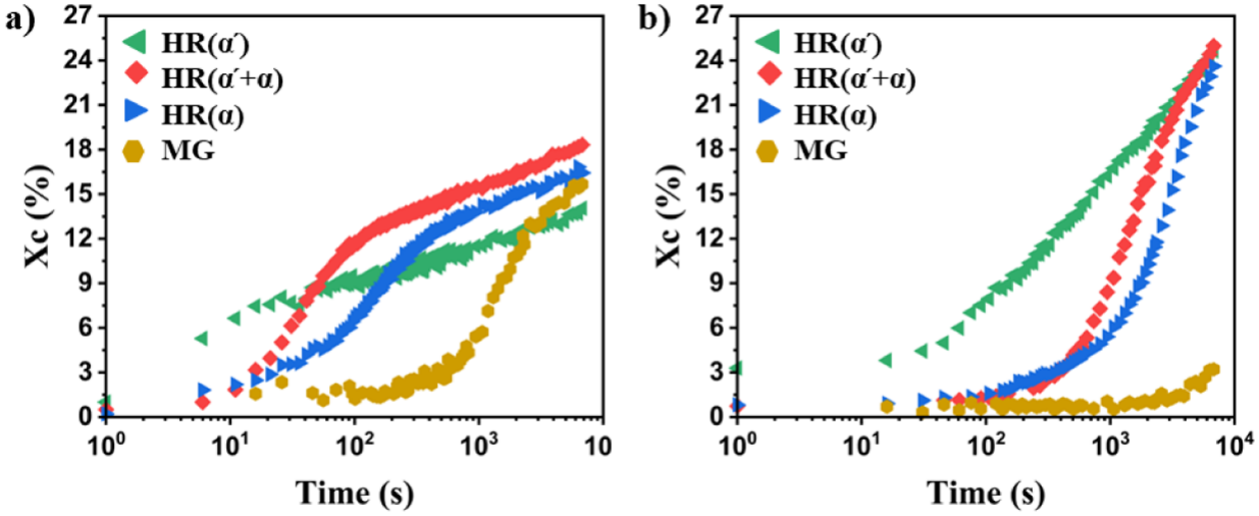
Time evolution of SC crystallinity during (re)­crystallization from different sources under isothermal conditions at a) 190 °C and b) 215 °C.

A quantitative comparison between the different samples reveals that SC crystals are formed earlier and more rapidly during homorecrystallization from the α′-type homocrystals, at both crystallization temperatures, an observation that is in line with previous findings.^31^ The homorecrystallization of the α-type exhibits slower kinetics compared to HR­(α′) and HR­(α′+α) at short times (t_c_ = 0–300 s), even though melt crystallization at 120 °C, resulting in HC­(α) leads to the formation of more HCs which can convert to SC crystals (Table S1). Imaging of the SC crystallization process via polarized optical microscopy, as shown in the movies in the Supporting Information, shows that most of the initial SC formation occurs at the location of the HC spherulites. Moreover, the smaller α′-type spherulites, which are known to melt and refold more easily than α spherulites,^50,67^ lead to a higher nucleation density. This suggests that not the overall crystallinity, but the presence of spherulite boundaries, which are known to melt first^70^ and can provide interface between L and D chains, dominate the crystallization kinetics.

Furthermore, the SC crystals already present in the HC­(α) sample may induce a further physical network effect that suppresses stereocomplexation at elevated temperatures. In contrast, melt crystallization at 80 and 100 °C, which corresponds to the formation of the α′ and α′+α types, respectively, does not result in SC crystal formation due to insufficient mobility for the conversion of paired helices to SC crystals at these low temperatures. As a result, there may be no or less suppression effect during homorecrystallization of the α′ and α′+α type HCs as compared to the HC­(α) samples. Finally, after 120 min, which we consider a practical upper limit for the crystallization process, a larger amount of SC crystals is produced during homorecrystallization of the α′+α type at both crystallization temperatures. Irrespective of the temperature, MG samples show a much slower crystallization kinetics as compared to HC samples. The slower crystallization kinetics of the MG sample can be attributed to its low nucleation density, as evidenced in Movie S1, combined with the lack of enhanced growth. This is in contrast to HC samples, for which PLLA and PDLA chains may be released in helical or other intermediate (partially ordered) configurations^29,71^ that speed up the crystallization process.

### Deconvolution of Primary and Secondary SC Crystallization Regimes

As discussed in the previous section, the evolution of the SC crystallinity versus time (Figure 8) suggests the presence of two distinct regimes, which can be indicated as primary and secondary crystallization. This two-stage crystallization process corresponds with the melting thermographs obtained after different times of SC crystallization (Figure 6c,d), wherein the high melting temperature corresponds to more stable crystals formed from the start of the crystallization and the lower one with less stable crystals generated during the later stages of crystallization. Similar to homorecrystallization, the growth of SC via mesophase growth also displays a secondary stage with a linear increase in crystallinity after an initial sigmoidal growth phase. Nevertheless, this does not result in a double melting peak, suggesting a different mechanism.

To accurately deconvolute the primary and secondary crystallization stages and derive valuable insights on the underlying mechanisms, an Avrami-type model is employed.^72,73^ Since the stereocomplex secondary crystallization stage only appears after a significant amount of primary crystallization has occurred, an integral Avrami model, developed for cases in which during the secondary crystallization stage crystalline zones grow from the primary crystallites, is selected.^74–76^ Eqs 4 and 5 formalize this model:^76^

4 $$X_{\mathrm{c‐total}}\left(t\right)=w_{1}X_{\mathrm{cP}}\left(t\right)+w_{2}X_{\mathrm{cS}}\left(t\right)$$

$$X_{\mathrm{c‐total}}\left(t\right)=\left[w_{1}\left(1-\exp\left(-k_{p}t^{n_{p}}\right)\right)+w_{2}\int_{0}^{t}X_{\mathrm{cP}}\left(t_{p}\right)\frac{\partial }{\partial t}\left(1-\exp\left(-k_{s}{\left(t-t_{p}\right)}^{n_{s}}\right)\right)dt_{p}\right]$$ 5

where *k*_p_ and *k*_s_ denote the rates of primary and secondary crystallization, respectively, and *n*_*p*_ and *n*_*s*_ are determined by the shape of the growing crystals. In this model, *w*_1_+*w*_2_ = 1, and each *w*_*i*_ indicates the relative crystallinity mass fraction at infinite time, thereby providing the relative importance of primary and secondary crystallization.

Figure 9 displays the deconvolution of the primary and secondary crystallinity contributions to the overall degree of crystallinity for the HR­(α′+α) at 190 and 215 °C using these equations. It is apparent that during the early stages of crystallization, the curves of the total crystallinity and primary crystallinity overlap, indicating that secondary crystallization is not significant during this stage. Moreover, there is a change in the crystallization kinetics during the secondary crystallization, which corresponds to a slowing down of the initial crystallization accompanied by the formation of a second crystalline structure.

**Figure 9:**
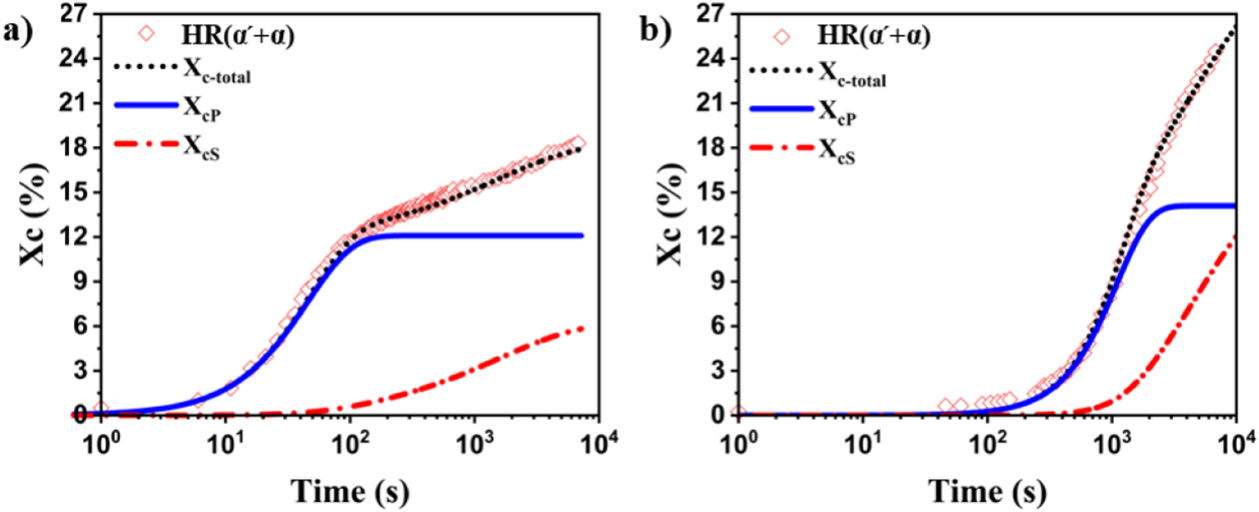
Evolution of the fitted experimental crystallinity, calculated primary (Χ_cP_) and secondary (Χ_cS_) crystallinity as a function of time for HR­(α′+α) at a) 190 °C and b) 215 °C.

All the parameters of eq 5 were determined by applying an iterative fitting procedure based on minimizing the least-squares error. The calculated Avrami model parameters and the half times of primary and secondary crystallization ( ${t_{p}}^{0.5}={\left(\ln2/k_{p}\right)}^{1/n_{p}}$ ) and ( ${t_{s}}^{0.5}={\left(\ln2/k_{s}\right)}^{1/n_{s}}$ ) are given in Table S2 for all the samples. Based on the half-time of crystallization, it can be observed that SC crystals tend to form at a faster rate at 190 °C. The Avrami index has often been utilized to determine the geometry of crystal growth and can provide valuable insights in the underlying mechanisms driving the process.^60^ The results indicate similar values of the primary Avrami index (*n*_*p*_) for the SC crystals grown from the mesophase at different crystallization temperatures namely 2.7 and 2.8 for T_sc_ = 190 °C and T_sc_= 215 °C, respectively. Since these values fall systematically in the same range (between 2 and 3), the SC growth mechanism by mesophase growth is assumed to be similar at both crystallization temperatures. A value near 3 in the primary crystallization step of MG samples, in accordance with Avrami theory, corresponds to crystallization with three-dimensional growth and instantaneous nucleation, as is commonly found in the literature.^60,77^ The observation of a single melting peak and its increase with crystallization time for MG samples in the DSC thermograms (Figure S10c and f) and the results obtained through optical microscopy experiments (Figure 4a and Movie S1) provide evidence supporting the assumption that stereocomplexation of the mesophase takes place through spherulitic growth, that is, a three-dimensional process.

Moreover, the results indicate that, at both the examined crystallization temperatures, the primary Avrami index values for SC crystals formed through homorecrystallization are lower as compared to those obtained for mesophase growth. Specifically, the lower values of n_p_ measured for the HR­(α′) indicate a predominant one-dimensional (1D) growth mode, which is not influenced by the SC crystallization temperature. However, at the same crystallization temperature, nearly equal n_p_ values are determined for the HR­(α) and HR­(α′+α) types, namely approximately 1 at T_sc_ = 190 °C, and approximately 2 at T_sc_ = 215 °C. This implies that SC crystals initially grow in one dimension at low T_sc_ and two-dimensional at high SC crystallization temperatures.

The same analysis has been reported by Na et al.^31^ based on the Avrami model using FTIR results. They found that annealing and crystallization temperatures strongly influence the Avrami exponent n in 1:1 PLLA/PDLA blends. Specifically, they observed one-dimensional and three-dimensional growth of SC crystals in blends annealed at 80 and 120 °C (HC­(α′) and HC­(α)), respectively, with a crystallization temperature of 200 °C. However, they mentioned that at 190 °C, two-dimensional growth of SC crystals was observed in the blend annealed at 120 °C, with an Avrami exponent n of 2.07 which is different from the current results. Multiple factors may be the reason for this different observation such as the different molecular weight of the samples, the sample preparation and the thermal protocol used for the homorecrystallization process. In addition, they used the classical Avrami equation with one single Avrami exponent to capture both crystallization stages, which may result in a different value for n.

In addition to n_p_, the secondary Avrami index (*n*_*s*_) is found to be close to 1 for both T_sc._ The n_s_≈ 1 (one-dimensional crystal growth) is often linked to processes such as lamellar thickening, crystallite impingement, perfection of crystals (crystallization between lamellae), or the stacking of lamellae, particularly when primary crystallization adopts a spherulitic growth mode.^75,78–80^ In the MG samples, the single melting peaks slightly shift to higher temperatures with increasing crystallization time at both the crystallization temperatures, indicating the involvement of a certain perfectioning mechanism during secondary crystallization. The results also show that the n_s_ values are smaller than their *n*_*p*_ counterparts and are lower than one for all SC crystallization processes through homorecrystallization at both T_sc_ (0.65 < n_s_ < 0.69 for T_sc_ = 190 °C and 0.82 < n_s_ < 0.89 for Tsc = 215 °C). For the homorecrystallization process, the appearance of multiple melting peaks and their shift to higher temperatures with increasing crystallization time indicates a different perfectioning mechanism compared to that in the mesophase growth process.

In general, it is clear that different HC crystal sources or mesophases as well as temperature conditions control the kinetics and microstructure of SC crystal formation from the cold state. Figure 3 shows that the different HC crystal sources not only differ in unit cell structure on the Angström scale or lamellar features on the nanometer scale, but also in spherulite size on the micrometer level. As such, the HC­(α′) result in an initially faster SC crystallization, then being overtaken by the HC­(α′+α) type, despite the higher initial crystallinity of the HC­(α) type. Melting is initiated at the crystal boundaries,^70^ and polymer chains may be released in helical or other intermediate (partially ordered) configurations^29,71^ that stimulate intermolecular interactions and thus can drive faster recrystallization. Hence, the faster melting^50,67^ and smaller spherulite sizes of the HC (α′) could cause the faster SC crystallization. However, Fujita et al.^29^ show that SC crystals start to form at the interfaces between the L and D lamellae. These L and D lamellae are shown to be present adjacent to each other within a single HC spherulite.^29^ Therefore, a quantitative link between the HC microstructure and SC crystallization kinetics would require the quantification of the amount of interface between L and D lamellae. Although it could be assumed in first approximation that the L and D domain sizes correspond to the scale of the HC lamellae, for which lamellar thickness and lateral dimension (spherulite radius) are given in Figures S3 and 3, an accurate determination of this amount of interface is faced with several challenges. First, HC lamellae can contact each other in either a flat-on or edge-on orientation, and the inner lamellae within spherulites can twist or bend into entirely different orientations.^81,82^ Second, even if phase separation between the L and D type chains is a result of crystallization and not already initiated by the temperature change, it cannot be excluded that L and D domains group multiple L or D lamellae. Hence, estimation of these domain sizes from the lamellar and spherulite dimensions is not necessarily accurate. Finally, the combination of the small length scales involved and limited techniques that have discriminating capability between l- and d-enantiomers that only differ in their chirality, hampers a direct characterization of these domains. Hence, a detailed quantification of the amount of L-D interfaces is considered very relevant to unravel the intricate relations between HC microstructure and SC kinetics and microstructure, but requires further developments.

## Conclusion

By using in situ SAXS/WAXD and calorimetry techniques resolved in both time and temperature, we investigated the crystallization and structural formation kinetics of stereocomplex crystals generated through melting and recrystallization of homocrystals or mesophase growth. It was demonstrated that melting and recrystallization of homocrystals can facilitate stereocomplex formation at both the crystallization temperatures, i.e., T_sc_= 190 and 215 °C. The homorecrystallization of a mixture of α′ and α-type HCs can lead to the formation of a higher amount of stereocomplex crystals at both of the crystallization temperatures. The melting recrystallization of HCs at the lower temperature results in the generation of small stereocomplex crystals with nodular shapes. On the other hand, at higher temperatures, the homorecrystallization process leads to the formation of coarser lamellar structures, whereas the mesophase growth mechanism leads to the formation of large stereocomplex crystals with spherulitic shapes. For the different types of initial structures (mesophase, α, α′ and α′+α), the SC crystallization is a two-stage process, whereby stable SC crystals are generated during primary crystallization, followed by the formation of more imperfect lamellae that undergo perfectioning in the secondary crystallization stage. This study provides important insights into the crystallization behavior of stereocomplex crystals from the cold state, elucidating how their size, quantity, and morphology can be controlled. The findings of this investigation are anticipated to facilitate the development of PLA-based materials with tailored properties.

## Supplementary Material











## Notes

Figures: (1) Enthalpy evolution during isothermal melt crystallization at *T*_c_. (2) Time-resolved WAXD profiles during melt crystallization at *T*_c_. (3) Crystallinity evaluation of different homocrystal types using WAXD and correlation functions from SAXS. (4) AFM phase images of different HC. (5) Thermal protocol used for generating SC crystals at different crystallization temperatures and DSC results during cooling. (6) POM micrographs of stereocomplex crystals formed from different sources and at different isothermal crystallization temperatures. (7) AFM phase images of SC crystals formed from different sources and at different isothermal crystallization temperatures. (8) Deconvolution of the WAXD profile into the amorphous and crystalline contributions. (9) DSC scans at different heating rates after stereocomplex crystallization. (10) Evolution of the melting behavior after stereocomplex crystallization for various times and for different sources. (11) Evolution of 1D-WAXD patterns during SC crystallization from different sources under isothermal conditions. (12) Evolution of crystallinity during isothermal stereocomplex crystallization at different temperatures using DSC. Tables: (1) Fitting results performed on the WAXD profile for different types of homocrystals. (2) The fitted parameters of the integral Avrami model (PDF)
POM of MG crystallization (MP4)
POM of HR­(α) crystallization (MP4)
POM of HR­(α') crystallization (MP4)
POM of HR­(α'+α) crystallization (MP4)
