# Supplementary material for: Formation of PLA Stereocomplex Crystals through Homorecrystallization and Mesophase Growth Mechanisms
Source: Macromolecules. 2025 May 9;58(11):5651–64. doi: 10.1021/acs.macromol.4c02836 (PMC12164531; doi:10.1021/acs.macromol.4c02836)
Supplement: Supplementary file 1 [file ma4c02836_si_001.pdf]

Supporting Information:

Formation of PLA stereocomplex crystals through homo-recrystallization and mesophase growth mechanisms

Hamid Ahmadi[a], *Stan F.S.P. Looijmans*[a], *Marc P. F. H. L. van Maris*[b], *Pauline Schmit*[c], *Siavash Maraghechi*[d], *Patrick D. Anderson*[a], *Ruth Cardinaels*[*a,e]

[a] Processing and Performance of Materials, Department of Mechanical Engineering, Eindhoven University of Technology, P.O. Box 513, 5600 MB Eindhoven, The Netherlands

*R.M.Cardinaels@tue.nl

[b] Multi-Scale Lab, Mechanics of Materials, Department of Mechanical Engineering, Eindhoven University of Technology, P.O. Box 513, 5600 MB Eindhoven, The Netherlands

[c] Department of Chemical Engineering and Chemistry, Eindhoven University of Technology, P.O. Box 513, 5600 MB Eindhoven, The Netherlands

[d] Applied Mechanics, Department of Built Environment, Eindhoven University of Technology, P.O. Box 513, 5600 MB Eindhoven, The Netherlands

[e] Soft Matter, Rheology and Technology, Department of Chemical Engineering, KU Leuven, Celestijnenlaan 200J, 3001 Leuven, Belgium *Ruth.Cardinaels@kuleuven.be

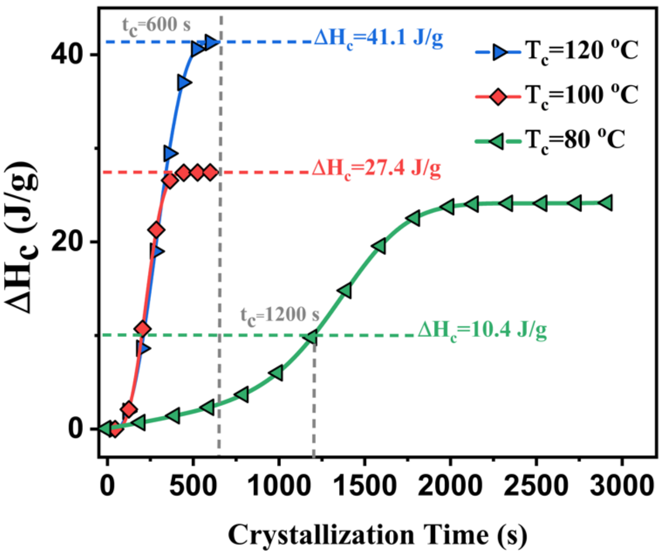

Figure S1. The enthalpy evolution during isothermal melt crystallization at 80, 100 and 120 °C, crystallization enthalpies after annealing times of 20, 10, and 10 min, respectively.

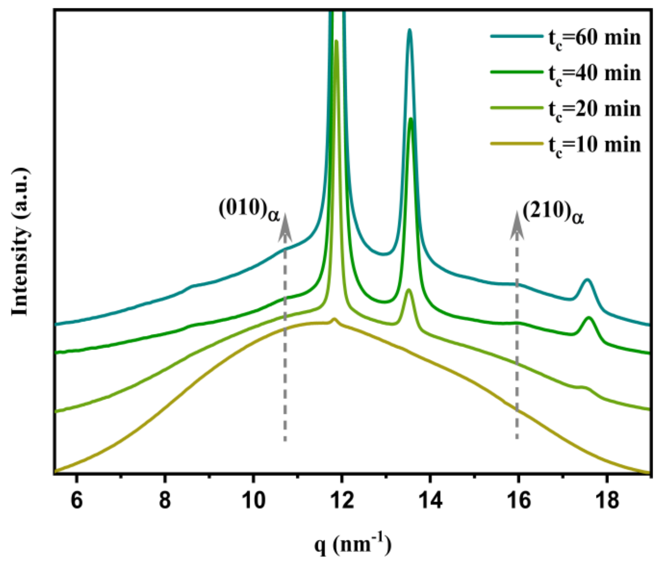

Figure S2. Time-resolved WAXD profiles during melt crystallization at 80 °C.

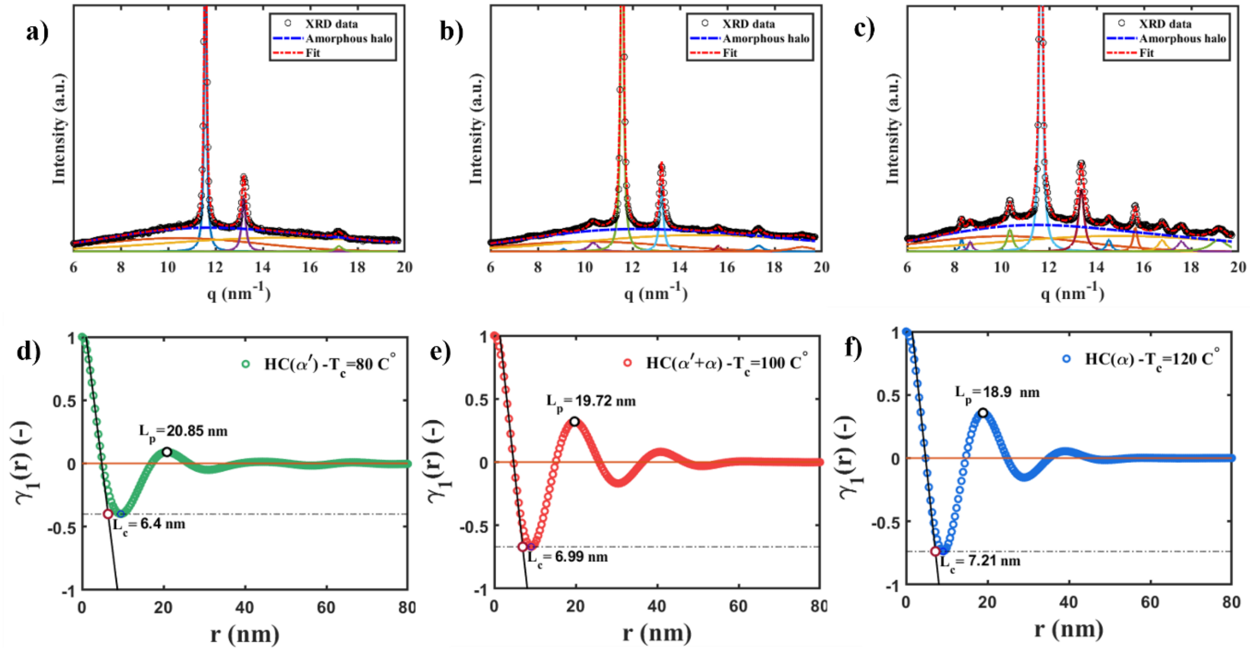

Figure S3. Crystallinity evaluation of different homocrystal types by peak deconvolution from WAXD profiles, a) HC(α′), b) HC(α′+α), c) HC(α). Correlation functions calculated from the SAXS profiles, d) HC(α′), e) HC(α′+α), f) HC(α).

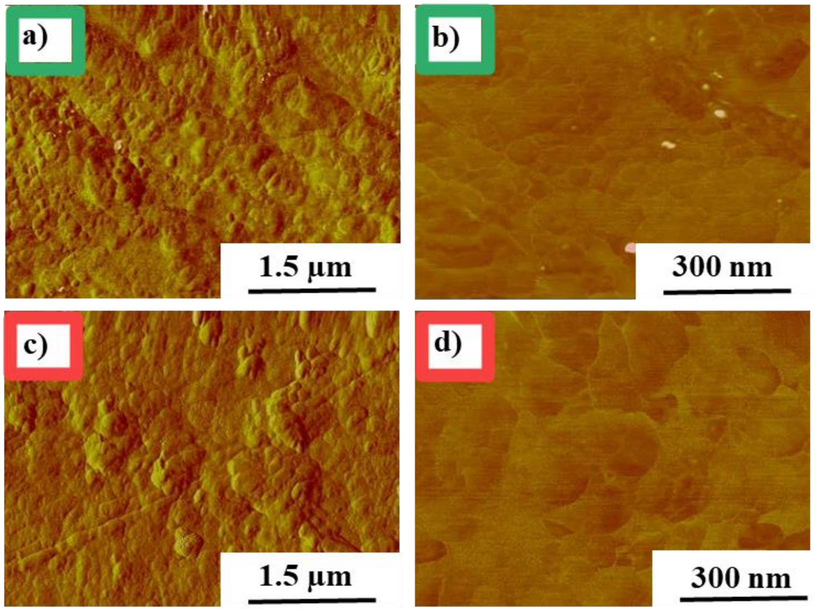

Figure S4. AFM phase images of HC(α′) prepared at 80 °C and 1200 s at different magnifications (a,b), HC(α′+α) prepared at 100 °C and 600 s at different magnifications (c,d).

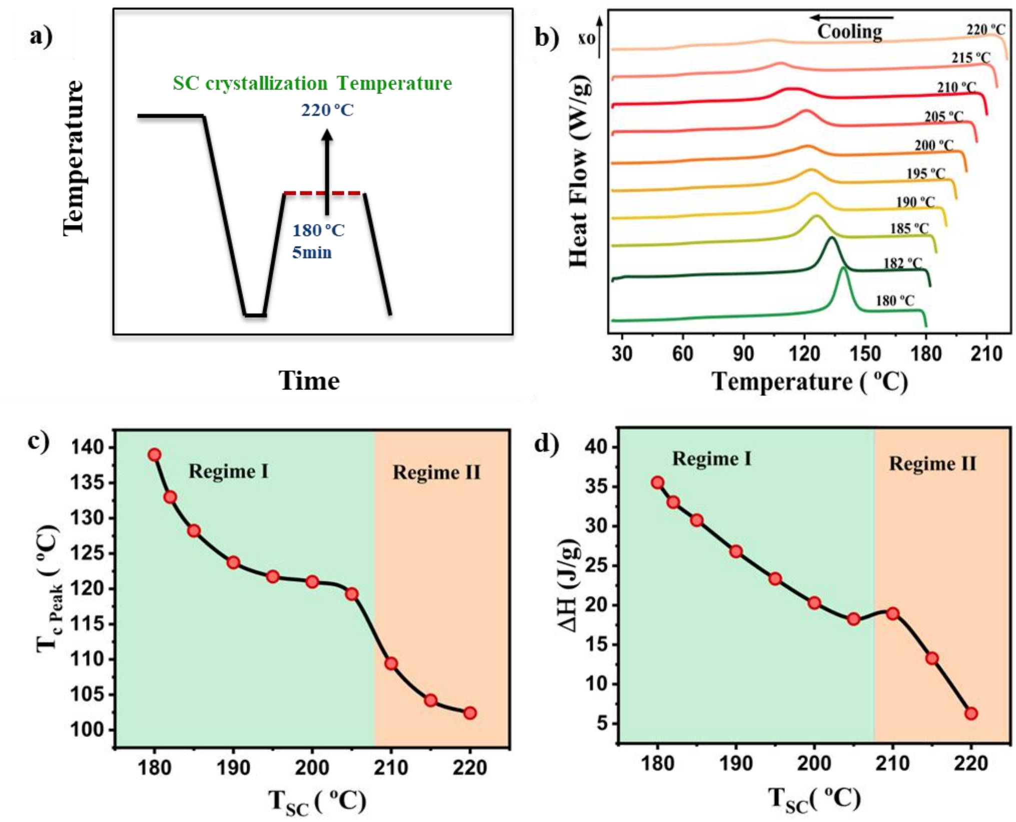

Figure S5. a) Thermal protocol used for generating SC crystals at different crystallization temperatures, b) DSC scans during cooling from the indicated T_{sc} in the range of 180-220 °C, c) peak crystallization temperature (T_{cPeak}) as a function of T_{sc}, d) Non-isothermal specific enthalpy of crystallization versus T_{sc}.

Note: To determine suitable values for the SC cold crystallization temperatures, in terms of reaching sufficient difference in chain mobility, we first assess differences in the formed SC crystals by evaluating the non-isothermal crystallization of the blend after cooling from the SC crystallization temperature. This is a sensitive probing method because the SC crystals formed at T_{sc} can alter the crystallization behavior of the system at lower temperatures, by affecting both homo and SC crystallization due to the nucleating effect of SC crystals in PLLA/PDLA blends[1]. This phenomenon can be observed in non-isothermal crystallization during cooling, where alterations in the crystallization temperature and quantity of crystals caused by stereocomplexation at different T_{sc} can be evaluated. To achieve this a specific thermal protocol (Figure S5a) is used that differs from the one in Figure 1a by the omission of step 1. First, the thermal history of the sample is removed at 260°C. After that the sample is cooled to room temperature at 50 °C/min which is sufficient to prevent crystallization during the cooling step [2, 3] and then heated at a rate of

10°C/min to a temperature denoted as T_{sc}, ranging from 180 to 220°C, where only SC crystals are formed, and kept at this temperature for 5 minutes. After this step, we assess the impact of SC crystals generated at different T_{sc} on non-isothermal crystallization during the cooling step at a cooling rate of 10 °C/min.

Table S1. Representative fitting results from the WAXD profile for different types of HCs.

**Sample name**	**T_c (°C)**	**t_c (s)**	**$X_{c,HC}$ (%)**	**L_p (nm)**	**L_c (nm)**	**$X_{c,Sc}$(%)**
HC(α′)	80	1200	25.9	20.8	6.4	0
HC(α′+α)	100	600	35.7	19.7	6.9	0
HC(α)	120	600	38.4	18.9	7.2	3.2

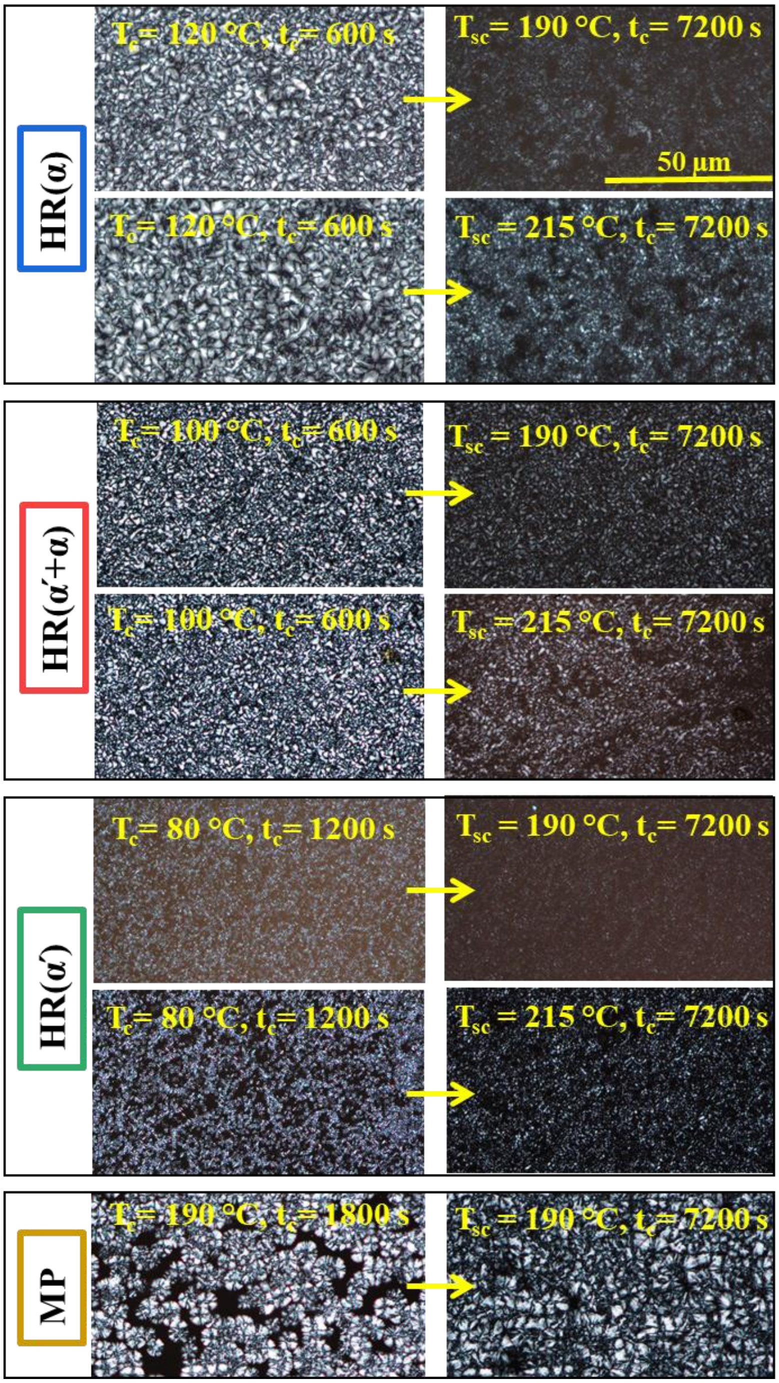

Figure S6. POM micrographs of stereocomplex crystals formed from different sources and at different isothermal crystallization temperatures. The images are labeled with the corresponding crystallization mechanism, as well as the temperature and time parameters used. The scales of all POM images are the same.

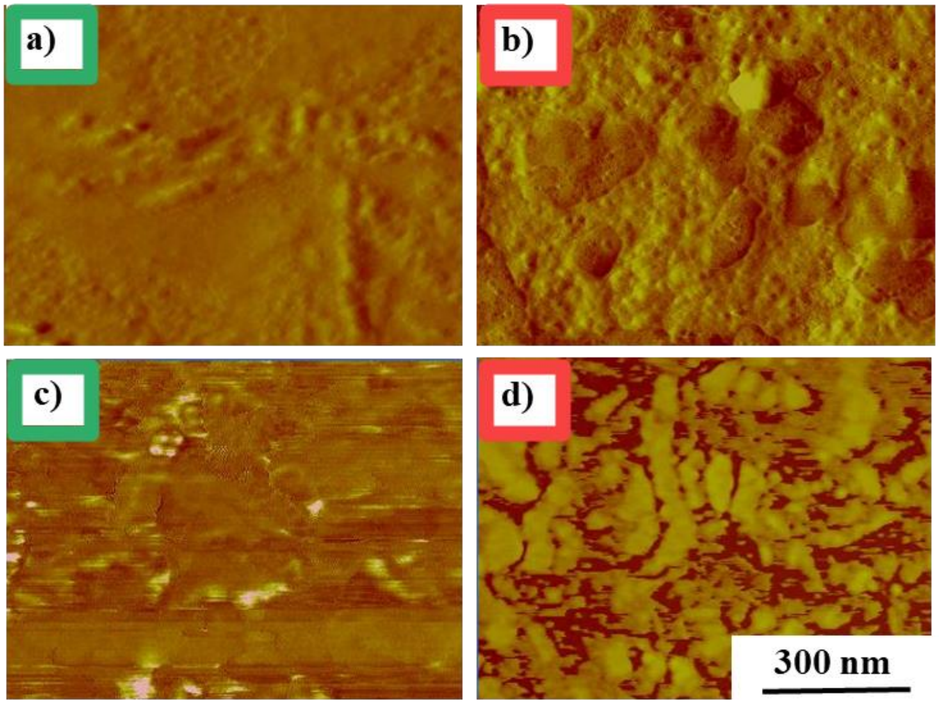

Figure S7. AFM phase images of SC crystals formed from different sources and at different isothermal crystallization temperatures, a) HR(α′) at 190 °C after tc=120 min , b) HR(α′+α) at 190 °C after tc=120 min, c) HR(α′) at 215 °C after tc=120 min, d) HR(α′+α) at 215 °C after tc=120 min, The scales of all AFM images are the same.

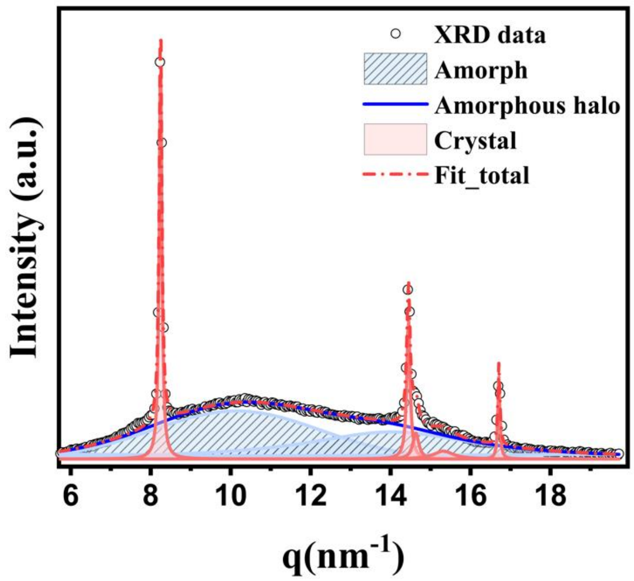

Figure S8. Deconvolution of the HR(α) WAXD profile into the amorphous and crystalline contributions.

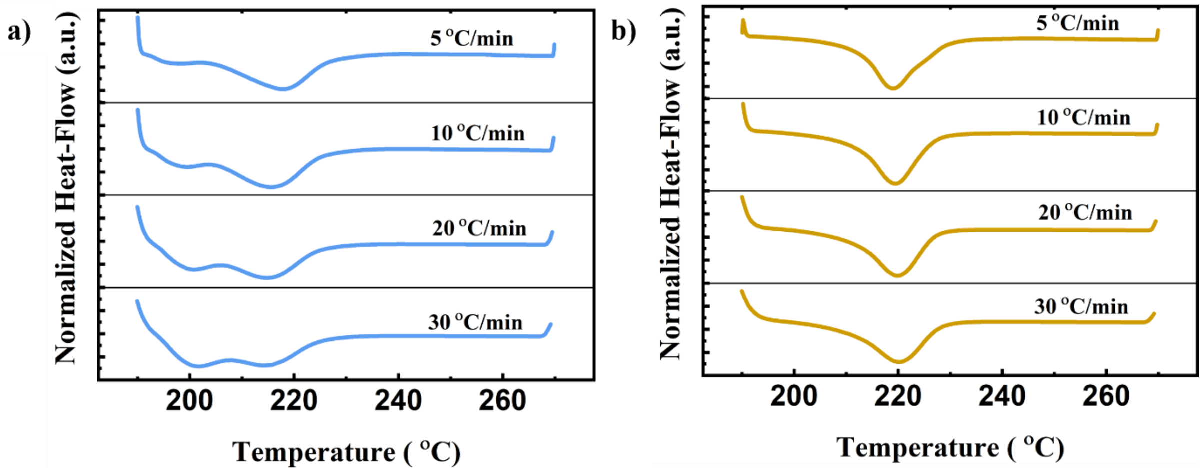

Figure S9. DSC scans at different heating rates after SC crystallization for 120 minutes at 190 °C. a) HR(α), b) MG.

.

Table S2. The parameters of the integral Avrami model obtained by fitting equation 5 to the experimental data.

Fitting parameter	HR(α′)- T_{SC} =190 °C	HR(α′+α)- T_{SC} =190 °C	HR(α)- T_{SC} =190 °C	MG- T_{SC} =190 °C
n_p	1.01	1.21	1.24	2.7
k_p	0.16	0.0098	0.0021	3.14E-09
w_1	0.54	0.66	0.66	0.71
$t_n^{0.5}$	4.26	33.77	107.43	1231.20
n_s	0.65	0.68	0.69	0.94
k_s	0.0098	0.0064	0.0065	0.00065
w_2	0.46	0.34	0.34	0.29
$t_s^{0.5}$	700.70	961.10	849.13	1664.11
**Fitting parameter**	**HR(α′)-T_{SC}=215 °C**	**HR(α′+α)- T_{SC} =215 °C**	**HR(α)- T_{SC} =215 °C**	**MG- T_{SC} =215 °C**
n_p	1.03	1.65	1.69	2.8
k_p	0.0041	9.55E-06	2.33E-06	8.1E-12
w_1	0.44	0.48	0.51	0.77
$t_n^{0.5}$	145.59	882.98	1732.76	8024.30
n_s	0.89	0.83	0.82	0.97
k_s	0.00071	0.00068	0.00066	0.000083
w_2	0.56	0.52	0.49	0.23
$t_s^{0.5}$	2285.93	4897.43	4836.03	11041.77

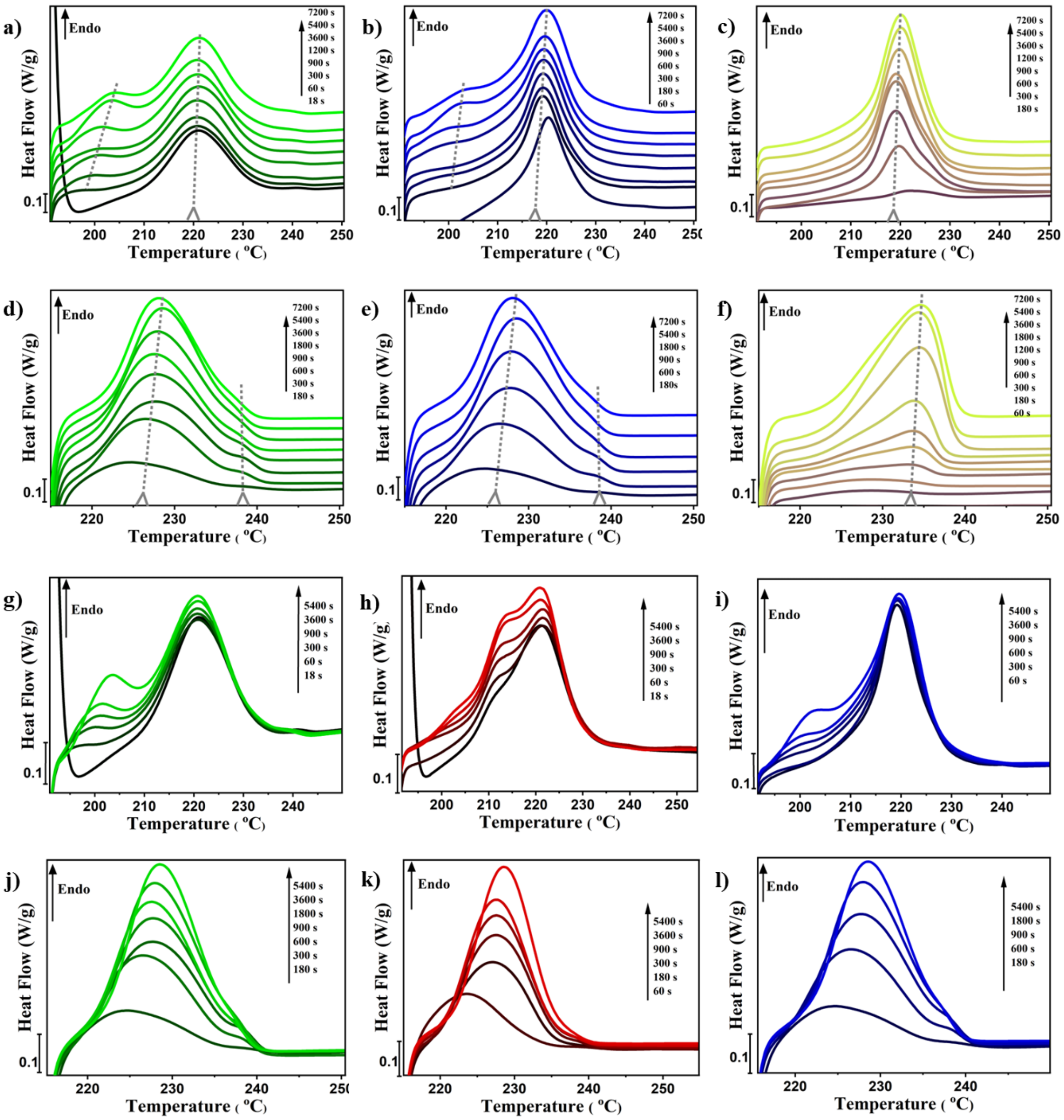

Figure S10. Evolution of the melting behavior after crystallization for various times during heating at 10.0 °C/min. Plots a to f are vertically shifted for clarity, plots g to l are presented without vertical shifts to enhance the visualization of peak evolution. a) HR(α′) at 190 °C, b) HR(α) at 190 °C, c) MG at 190 °C, d) HR(α′) at 215 °C, e) HR(α) at 215 °C, f) MG at 215 °C, g) HR(α′) at 190 °C, h) HR(α′+α) at 190 °C, i) HR(α) at 190 °C, j) HR(α′) at 215 °C, k) HR(α′+α) at 215 °C, l) HR(α) at 215 °C.

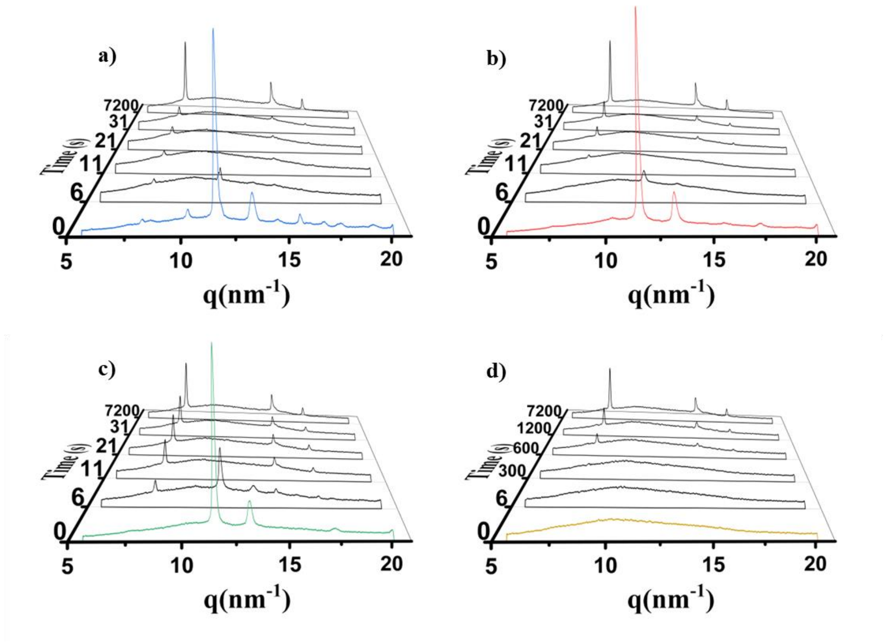

Figure S11. Evolution of 1D-WAXD patterns during SC crystallization from different sources under isothermal conditions at 190 ºC. a) HR(α), b) HR(α′+α), c) HR(α′), d) MG.

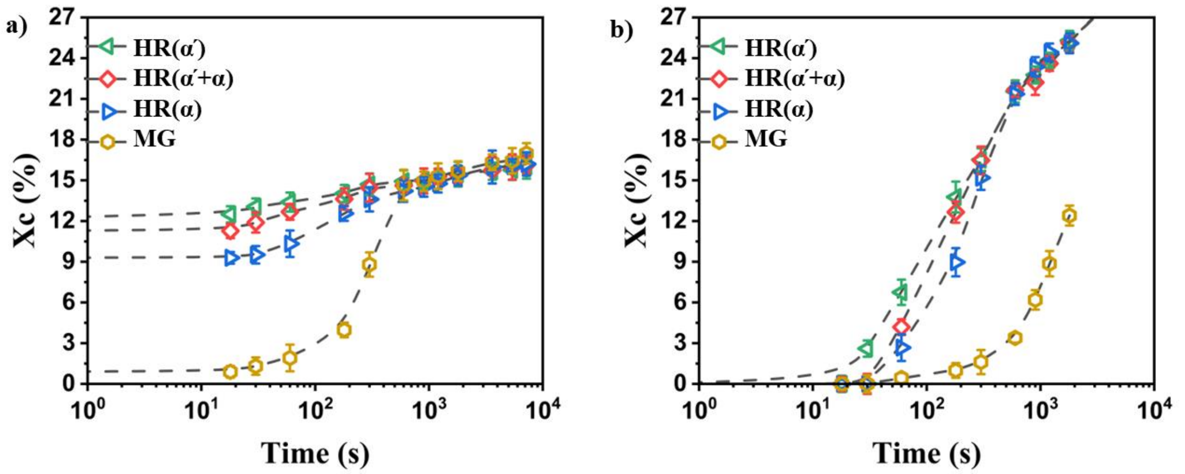

Figure S12. Evolution of crystallinity under isothermal SC crystallization at a) 190 ºC, and b) 215 ºC determined from calorimetric measurements. The dashed lines are plotted to guide the eyes.

Note: the quantity of SC crystals is estimated using $X_{c,sc} = \Delta H_{m,sc} / \Delta H_{m,sc}^*$, where $\Delta H_{m,sc}$ is the heat of fusion experimentally obtained for a given sample in the relevant temperature range after different crystallization times at 190 ˚C and 215 ˚C (Figure 6c,d and Figure S10), and $\Delta H_{m,sc}^* = 142$ J.g^{-1} is the theoretical melting enthalpy of a perfect stereocomplex crystal.[4, 5]

References

1. Wei, X. F.; Bao, R. Y.; Cao, Z. Q.; Yang, W.; Xie, B. H.; Yang, M. B., Stereocomplex Crystallite Network in Asymmetric PLLA/PDLA Blends: Formation, Structure, and Confining Effect on the Crystallization Rate of Homocrystallites. *Macromolecules* **2014,** *47* (4), 1439-1448.
2. Androsch, R.; Iqbal, H. N.; Schick, C. J. P., Non-isothermal crystal nucleation of poly (L-lactic acid). *Polymer* **2015,** *81*, 151-158.
3. Bai, J.; Wang, J.; Wang, W.; Fang, H.; Xu, Z.; Chen, X.; Wang, Z. J. A. S. C.; Engineering, Stereocomplex crystallite-assisted shear-induced crystallization kinetics at a high temperature for asymmetric biodegradable PLLA/PDLA blends. *ACS Sustainable Chemistry Engineering* **2016,** *4* (1), 273-283.
4. Tsuji, H.; Horii, F.; Nakagawa, M.; Ikada, Y.; Odani, H.; Kitamaru, R., Stereocomplex formation between enantiomeric poly (lactic acid) s. 7. Phase structure of the stereocomplex crystallized from a dilute acetonitrile solution as studied by high-resolution solid-state carbon-13 NMR spectroscopy. *Macromolecules* **1992,** *25* (16), 4114-4118.
5. Fischer, E.; Sterzel, H. J.; Wegner, G., Investigation of the structure of solution grown crystals of lactide copolymers by means of chemical reactions. *Kolloid-Zeitschrift und Zeitschrift für Polymere* **1973,** *251* (11), 980-990.
